# Lipid metabolism–related lncRNA *SLC25A21‐AS1* promotes the progression of oesophageal squamous cell carcinoma by regulating the NPM1/c‐Myc axis and *SLC25A21* expression

**DOI:** 10.1002/ctm2.944

**Published:** 2022-06-23

**Authors:** Yu Liu, Chunxiang Li, Lingling Fang, Liyu Wang, Hengchang Liu, He Tian, Yujia zheng, Tao Fan, Jie He

**Affiliations:** ^1^ Department of Thoracic Surgery National Cancer Center/National Clinical Research Center for Cancer/Cancer Hospital Chinese Academy of Medical Sciences and Peking Union Medical College Beijing China; ^2^ Department of Colorectal Surgery National Cancer Center/Natbibional Clinical Research Center for Cancer/Cancer Hospital Chinese Academy of Medical Sciences and Peking Union Medical College Beijing China

**Keywords:** high‐fat diets, long non‐coding RNA, oesophageal squamous cell carcinoma, palmitic acid

## Abstract

**Background:**

Obesity alters metabolic microenvironment and is thus associated with several tumours. The aim of the present study was to investigate the role, molecular mechanism of action, and potential clinical value of lipid metabolism–related long non‐coding RNA (lncRNA) *SLC25A21‐AS1* in oesophageal squamous cell carcinoma (ESCC).

**Methods:**

A high‐fat diets (HFDs)‐induced obesity nude mouse model was established, and targeted metabolomics analysis was used to identify critical medium‐long chain fatty acids influencing the growth of ESCC cells. Transcriptomic analysis of public dataset GSE53625 confirmed that lncRNA *SLC25A21‐AS1* was a lipid metabolism–related lncRNA. The biological function of lncRNA *SLC25A21‐AS1* in ESCC was investigated both in vivo and in vitro. Chromatin immunoprecipitation(ChIP)assay, RNA‐pull down, mass spectrometry, co‐IP, and RNA IP(RIP) were performed to explore the molecular mechanism. Finally, an ESCC cDNA microarray was used to determine the clinical prognostic value of *SLC25A21‐AS1* by RT‐qPCR.

**Results:**

Palmitic acid (PA) is an important fatty acid component of HFD and had an inhibitory effect on ESCC cell lines. LncRNA *SLC25A21‐AS1* expression was downregulated by PA and associated with the proliferation and migration of ESCC cells in vitro and in vivo. Mechanistically, *SLC25A21‐AS1* interacted with nucleophosmin‐1 (NPM1) protein to promote the downstream gene transcription of the c‐Myc in the nucleus. In the cytoplasm, *SLC25A21‐AS1* maintained the stability of *SLC25A21* mRNA and reduced the intracellular NAD^+^/NADH ratio by influencing tryptophan catabolism. Finally, we demonstrated that high expression of *SLC25A21‐AS1* promoted resistance to cisplatin‐induced apoptosis and was correlated with poor tumour grade and overall survival.

**Conclusions:**

HFD/PA has an inhibitory effect on ESCC cells and *SLC25A21‐AS1* expression. *SLC25A21‐AS1* promotes the proliferation and migration of ESCC cells by regulating the NPM1/c‐Myc axis and *SLC25A21* expression. In addition, lncRNA *SLC25A21‐AS1* may serve as a favourable prognostic biomarker and a potential therapeutic target for ESCC.

## Graphical abstract

1. Identified a lipid metabolism related lncRNA SLC25A21‐AS1 which can be downregulated by HFD/PA.
2. LncRNA SLC25A21‐AS1 promotes proliferation and migration of ESCC cells through the NPM1/c‐Myc axis and elevation of SLC25A21 expression.
3. LncRNA SLC25A21‐AS1 is associated with overall survival and tumor grade.

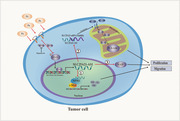

## 1 INTRODUCTION

Oesophageal squamous cell carcinoma (ESCC) is common in China and is characterized by a high degree of malignancy and poor prognosis.^1^, ^2^ ESCC patients do not present typical symptoms in the early stage, and most patients are diagnosed in the advanced stage. Understanding the pathogenic mechanism of ESCC will help to prevent, detect, and treat ESCC, and identification of specific molecules or risk factors associated with or involved in tumourigenesis and progression of ESCC is essential.

Obesity is a metabolic disease involving excessive body weight, and incidence of obesity has significantly increased in recent years.^3^ Obesity is associated with an increase in the risk for cancers.^3^ Abdominal obesity is associated with an increased risk for ESCC,^4^, ^5^ and good nutritional status is associated with a better prognosis for ESCC patients with obesity.^6^, ^7^ However, the exact role of obesity in the development and progression of ESCC is poorly understood. Hence, it is necessary to further explore the association between obesity and ESCC.

The macroenvironment and microenvironment of obesity influence fatty acid metabolism in cancer and cancer behaviour.^8^ Numerous adipocyte‐derived factors have been proposed to mediate these effects. Adipocyte‐derived fatty acids can act as the substrates for lipid synthesis and are involved in mitochondrial fatty acid oxidation in cancer cells through increased levels of a range of fatty acid metabolism proteins such as CPT1A or CPT1B.^9^, ^10^, ^11^ A high‐fat diet (HFD) is a major factor leading to hyperlipidaemia and obesity. HFD is associated with tumour growth by enhancing interleukin‐6 secretion or impairing CD8^+^T cell function.^12^, ^13^ Moreover, HFD causes metabolic alterations in tumour cells by promoting a Myc‐dependent transcriptional programme in prostate cancer.^14^ A previous study also showed that HFD induces the activation of adipose tissue macrophages (ATMs), which engulf tumour cells and suppress the peritoneal seeding of colorectal cancer.^15^ Palmitic acid (PA) is a common saturated fatty acid which is present at a high level in patients with hyperlipidaemia and is associated with several diseases, including tumours.^16^ PA has specific tumourigenic properties different from those of other saturated fatty acids and acts as a signalling molecule to influence tumour development.^16^ However, the effects of HFD and PA on the development and progression of ESCC and the underlying mechanisms, especially in patients with obesity, have not been investigated.

Long non‐coding RNAs (lncRNAs) are a type of RNA molecules with multiple functions that influence tumour progression, and some of lncRNAs are closely associated with the prognosis of ESCC.^17^, ^18^, ^19^, ^20^, ^21^ A series of cancer‐related lncRNAs are dysregulated in obesity, including ANRIL, H19, and HOTAIR.^22^, ^23^, ^24^, ^25^ These obesity‐related lncRNAs are also closely associated with tumour metabolism. For example, the downregulation of ANRIL is associated with altered expression of the genes related to glucose and fatty acid metabolism, which influence tumour progression.^25^ Our previous study showed that obesity has an effect on the tumour microenvironment and that several lncRNAs, including *SLC25A21‐AS1* (ENSG00000258708), are dysregulated in the ESCC tumour tissues.^26^ In addition, a previous study showed that lncRNA *SLC25A21‐AS1* promoted multidrug resistance in nasopharyngeal carcinoma.^27^ However, the effects of *SLC25A21‐AS1* on the progression of ESCC have not been elucidated. Therefore, the aim of the present study was to investigate the role, molecular mechanism of action, and potential clinical value of lipid metabolism–related lncRNA *SLC25A21‐AS1* in ESCC.

## 2 RESULTS

### 2.1 Identification of the inhibitory effect of HFD and PA

**FIGURE 1:**
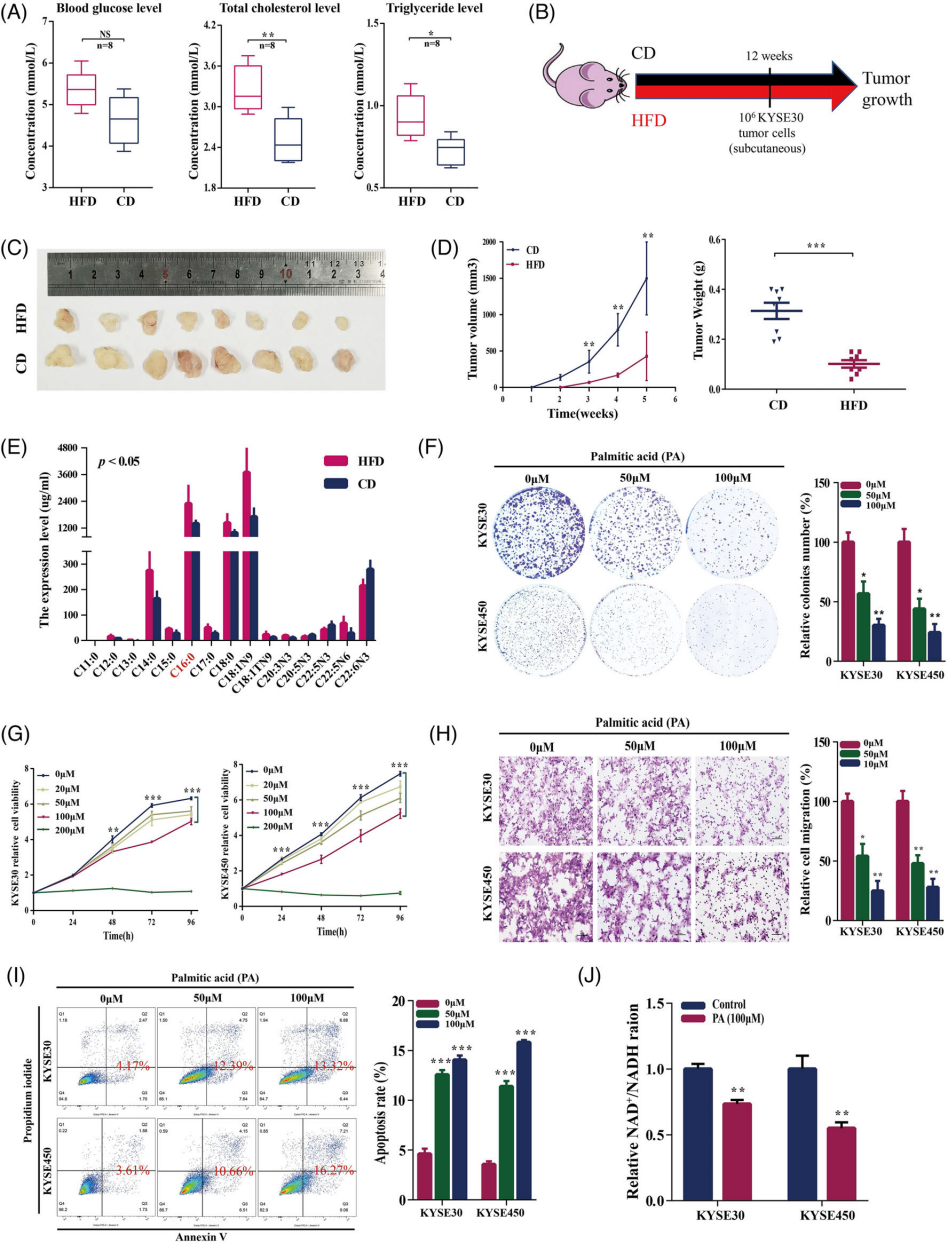
**The inhibitory effect of high‐fat diet (HFD) and palmitic acid (PA) on cell lines of oesophageal squamous cell carcinoma (ESCC), KYSE30, and KYSE450**. (A) Increased blood levels of glucose, total cholesterol, and triglyceride in the HFD group, compared with the control diet (CD) group. (B) The schematic diagram of the HFD‐induced obesity and xenograft tumour nude mice model. (C) The images and (D) volumes and weights of the xenograft tumours established in HFD and CD‐fed mice. (E) Significantly different levels of several medium and long chain fatty acids in xenograft tumour tissues between the HFD and CD groups as detected by targeted metabolomics. (F and G) The inhibitory effect of PA on colony formation and cell proliferation was detected by colony formation and CCK8 assays, respectively. (H and I) The inhibitory effect of different concentrations of PA (0, 50, and 100 μM) on migration and apoptosis of KYSE30 and KYSE450 cell lines as detected by Transwell assay and flow cytometry, respectively. (J) The 100‐μM PA reduced the relative NAD^+^/NADH ratio of KYSE30 and KYSE450 cell lines. The data are shown as mean ± standard deviation. *p < .05, **p < .01, and ***p < .001

The BALB/cA‐nu male mice were fed with HFD or control diet (CD) for 12 weeks. HFD‐fed mice had larger weight than CD‐fed mice (Figure S1A), and the severity of hyperlipidaemia was higher in the HFD group (Figure 1A). Subsequently, the ESCC cell line KYSE30 was used to establish a subcutaneous xenograft tumour model (Figure 1B). Mice were sacrificed after 5 weeks, and a decrease in the volume of the subcutaneous xenograft tumour tissues was detected in the HFD group (Figure 1C,D). Targeted metabolomics was used to explore the changes in the levels of medium long‐chain fatty acids in the samples of the ESCC cell xenograft tumour tissues of mice fed with HFD; the results indicated that the levels of several saturated and unsaturated fatty acids were significantly different between the HFD and CD groups (*p* < .05; Figure 1E). The levels of PA (C16:0; *p* = .022), stearic acid (C18:0; *p* = .023), and oleic acid (C18:1N9; *p* = .015) in the xenograft tumour tissues were significantly higher in the HFD group than those in the CD group. Three fatty acids, including PA, stearic acid, and oleic acid, at various concentrations (0, 20, 50, 100, and 200 μM) were used to stimulate KYSE30 and KYSE450 cells. The results of the colony formation and CCK‐8 assays showed that only PA had a significant inhibitory dose‐dependent effect on the ESCC cell lines (Figure 1F,G). In addition, stearic acid at low concentrations (20 and 50 μM) promoted the cell proliferation, and the cell proliferation were inhibited at high concentrations of stearic acid (100 and 200 μM, Figure S1B); oleic acid at all tested concentrations (20–200 μM) inhibited the cell proliferation (Figure S1C).

In addition, the exposure of KYSE30 and KYSE450 cells to PA suppressed the migration of the cells and induced apoptosis (Figure 1H,I). Previous studies have reported that plasma PA concentrations in healthy subjects range from 100 to 180 μM.^16^ To exclude the lipotoxic effect of PA accumulation, we treated Het‐1A human oesophagus epithelial cells with various concentrations of PA and detected the positive (20 and 50 μM) and negative effects (200 μM) on the proliferation (Figure S1D). We used various concentrations of PA to determine that the half‐maximal inhibitory concentration (IC50) was100 μM in both the KYSE30 and KYSE450 cell lines (Figure S1E). These results indicated that 100 μM was an effective inhibitory concentration of PA. To confirm the effect of PA on tumour growth, we treated CD‐fed mice with PA (10.26 mg/kg/day) by gavage and injected the animals with KYSE30 cells. The results indicated an inhibitory effect of this treatment on ESCC tumour growth (Figure S1F–H). Previous studies have shown PA influenced the NAD^+^/NADH ratio,^28^, ^29^ which is involved in tumour metabolism and homeostasis. The results of the assays of the NAD^+^/NADH ratio after 24 h of exposure to 100‐μM PA indicated a significant reduction in the NAD^+^/NADH ratio (Figure 1J).

### 2.2 LncRNA *SLC25A21‐AS1* is identified as a PA‐related lncRNA

**FIGURE 2:**
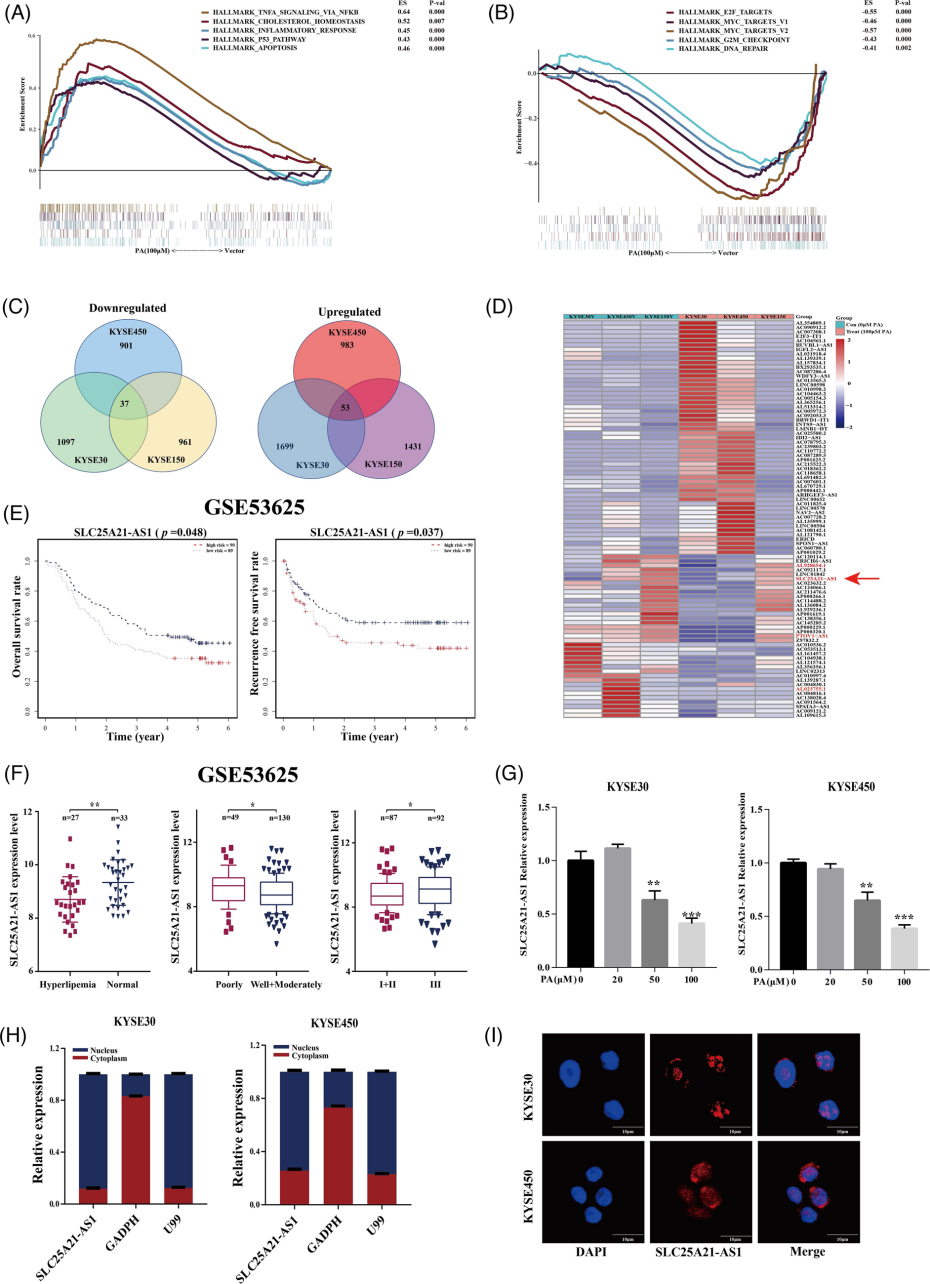
**Identification of long non‐coding RNA (lncRNA) *SLC25A21‐AS1* in oesophageal squamous cell carcinoma (ESCC) cells**. (A and B) The gene set enrichment analysis (GSEA) function enrichment analysis with RNA‐seq for the ESCC cells treated with 100‐μM palmitic acid (PA). The TNF signalling, inflammatory response, P53 pathway, and apoptosis were activated with PA treatment. The E2F targets, MYC targets, and DNA repair were inhibited with PA treatment. (C) A Venn diagram of the upregulated (right) and downregulated (left) lncRNAs under 100‐μM PA in three ESCC cell lines. (D) A heatmap of differentially regulated lncRNAs (i.e., with a fold chance >1 and a *p*‐value <.05). The four lncRNAs highlighted in red font indicate the lncRNAs intersected with the GSE53625 dataset. (E) *SLC25A21‐AS1* expression was associated with overall survival (OS) and RFS in the GSE53625 dataset. (F) Significant associations of *SLC25A21‐AS1* overexpression with clinical characteristics, including hyperlipidaemia, tumour differentiation grade, and TNM stage in the GSE53625 dataset. (G) *SLC25A21‐AS1* expression was reduced in ESCC cells treated with different PA concentrations (0–100 μM) as detected by RT‐qPCR. (H) The subcellular location of *SLC25A21‐AS1* was mainly in the nucleus as detected by RT‐qPCR. U99 and GAPDH are nuclear and cytoplasmic markers. (I) Enrichment of *SLC25A21‐AS1* in both the nucleus and cytoplasm (red) as detected by RNA fluorescence in situ hybridization (FISH). Diamidino‐2‐phenylindole staining cell nuclear is indicated in blue. The data are shown as mean ± SD. *p < .05; **p < .01; ***p < .001

RNA‐sequencing was performed in the KYSE30, KYSE450, and KYSE150 cell lines treated with 100‐μM PA for 24 h. The results of functional gene set enrichment analysis (GSEA) showed that TNF signalling, inflammatory response, the P53 pathway, and apoptosis were activated (Figure 2A), whereas E2F targets, MYC targets, and DNA repair were inhibited (Figure 2B). We identified several lncRNAs differentially expressed in patients with and without hyperlipidaemia in the GSE53625 dataset (Figure S2A). Subsequently, we identified 90 intersecting differentially expressed lncRNAs (i.e., with a fold chance >1 and *p*‐value <.05) after stimulation with 100‐μM PA in all three tested ESCC cell lines (Figure 2C). Furthermore, the levels of only four lncRNAs, including *AL023755.1, PTOV1‐AS1, AL928654.1*, and *SLC25A21‐AS1*, were significantly different according to the data of both RNA‐seq and database analysis (Figures 2D and S2B).

One identified lncRNAs, *SLC25A21‐AS1* was associated with overall survival (OS) and recurrence‐free survival (RFS) according to the data of the GSE53625 dataset (Figure 2E). A decrease in *SLC25A21‐AS1* expression was detected in patients with hyperlipidaemia and was associated with a good differentiation and a lower TNM stage (Figure 2F). The results of additional analysis showed that *SLC25A21‐AS1* expression was significantly reduced in patients with obesity (body mass index [BMI] > 28) in the GSE53625 dataset and in overweight patients (BMI: 24–27.9) in The Cancer Genome Atlas TCGA (Figure S2C). In addition, the expression of *SLC25A21‐AS1* was downregulated by the treatment with 50‐ and 100‐μM PA (Figure 2G), but not by stearic or oleic acids (Figure S2D,E). *SLC25A21‐AS1* was identified as a 1703‐bp full‐length cDNA sequence by a rapid amplification of cDNA ends (RACE) (Figure S2F; Table S3). *SLC25A21‐AS1* was mainly present in the nucleus according to the data of nuclear and cytoplasmic RNA fractionation and subsequent RT‐qPCR (Figure 2H), and this result was verified by RNA fluorescence in situ hybridization (FISH) (Figure 2I).

### 2.3 *SLC25A21‐AS1* promotes the progression of ESCC

**FIGURE 3:**
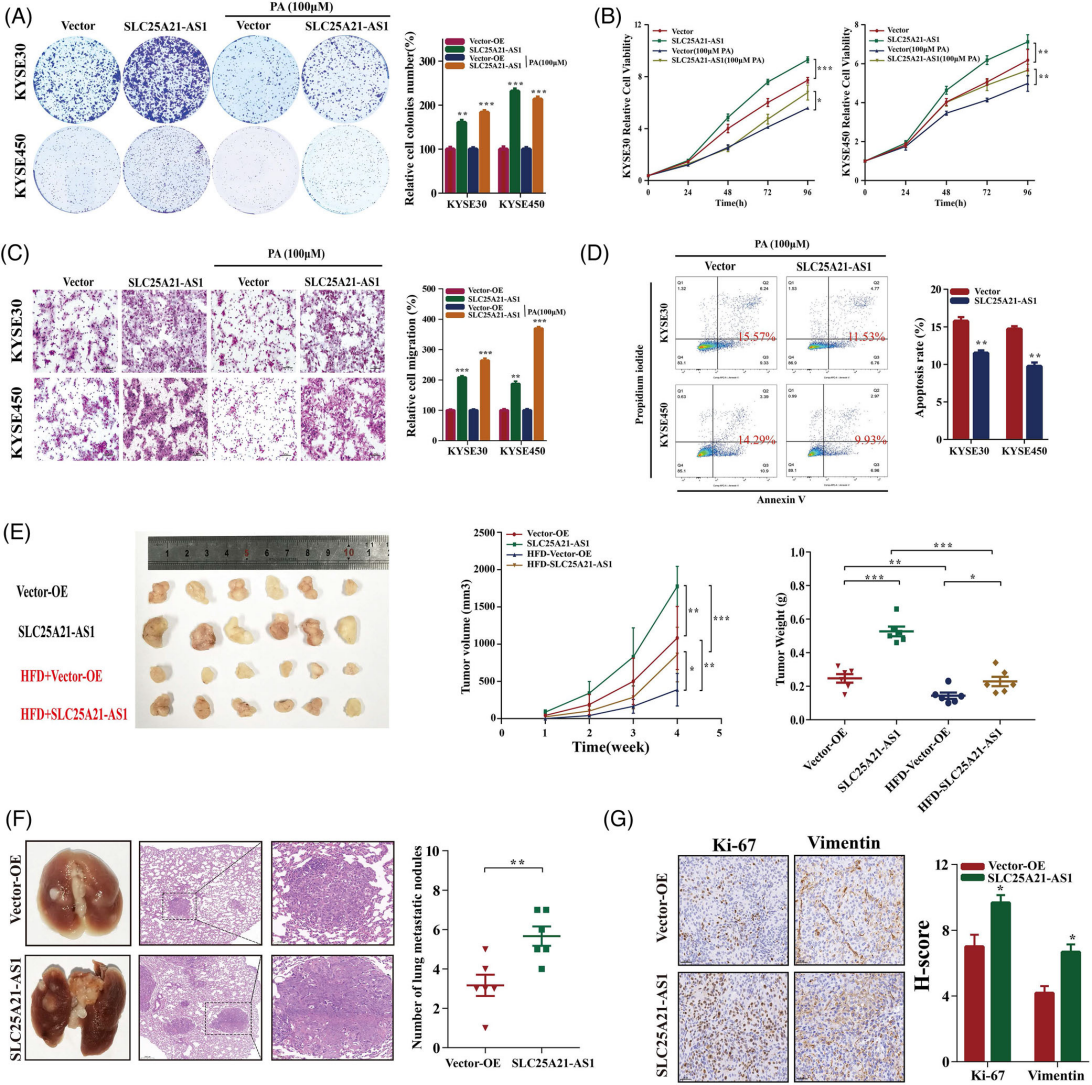
**Overexpression of *SLC25A21‐AS1* promotes oesophageal squamous cell carcinoma (ESCC) proliferation and migration in vitro and in vivo**. (A–C), Increased colony formation (A), cell proliferation (B), and migration (C) of ESCC cells overexpressing *SLC25A21‐AS1* versus vector control cells with or without palmitic acid (PA, 100 μM) treatment. The right panel for A and C shows the quantification results. (D) Increased apoptosis of ESCC cells overexpressing *SLC25A21‐AS1* versus vector control cells with PA (100 μM) treatment. (E) The images, volumes, and weights of xenograft tumours established by *SLC25A21‐AS1*‐overexpressing and control KYSE30 cells in BALB/c nude mice fed with a control diet (CD) and high‐fat diet (HFD), respectively. (F) Representative images of the gross lesion in lung tissues (left of the left panel) and haematoxylin and eosin staining of metastatic nodules in the lungs from the *SLC25A21‐AS1*‐overexpressing and vector groups (right of the left panel). The right panel shows the quantification results of metastatic nodule numbers. (G) Ki67 and vimentin immunostaining of tumour samples from the *SLC25A21‐AS1*‐overexpressing and vector groups. The right panel shows the quantification results. *p < .05, **p < .01, and ***p < .001

*SLC25A21‐AS1* overexpression promoted cell proliferation and reversed the inhibitory effect of PA (100 μM) (Figures 3A,B and S3). Additionally, *SLC25A21‐AS1* overexpression promoted the migration, induced resistance to 100‐μM PA‐induced apoptosis (Figure 3C,D), and enhanced tumour growth and nodular metastasis in animal experiments (Figure 3E,F). The results of immunohistochemical (IHC) analysis showed that Ki67 and vimentin were expressed at high levels in *SLC25A21‐AS1*‐overexpressing cells (Figure 3G).

**FIGURE 4:**
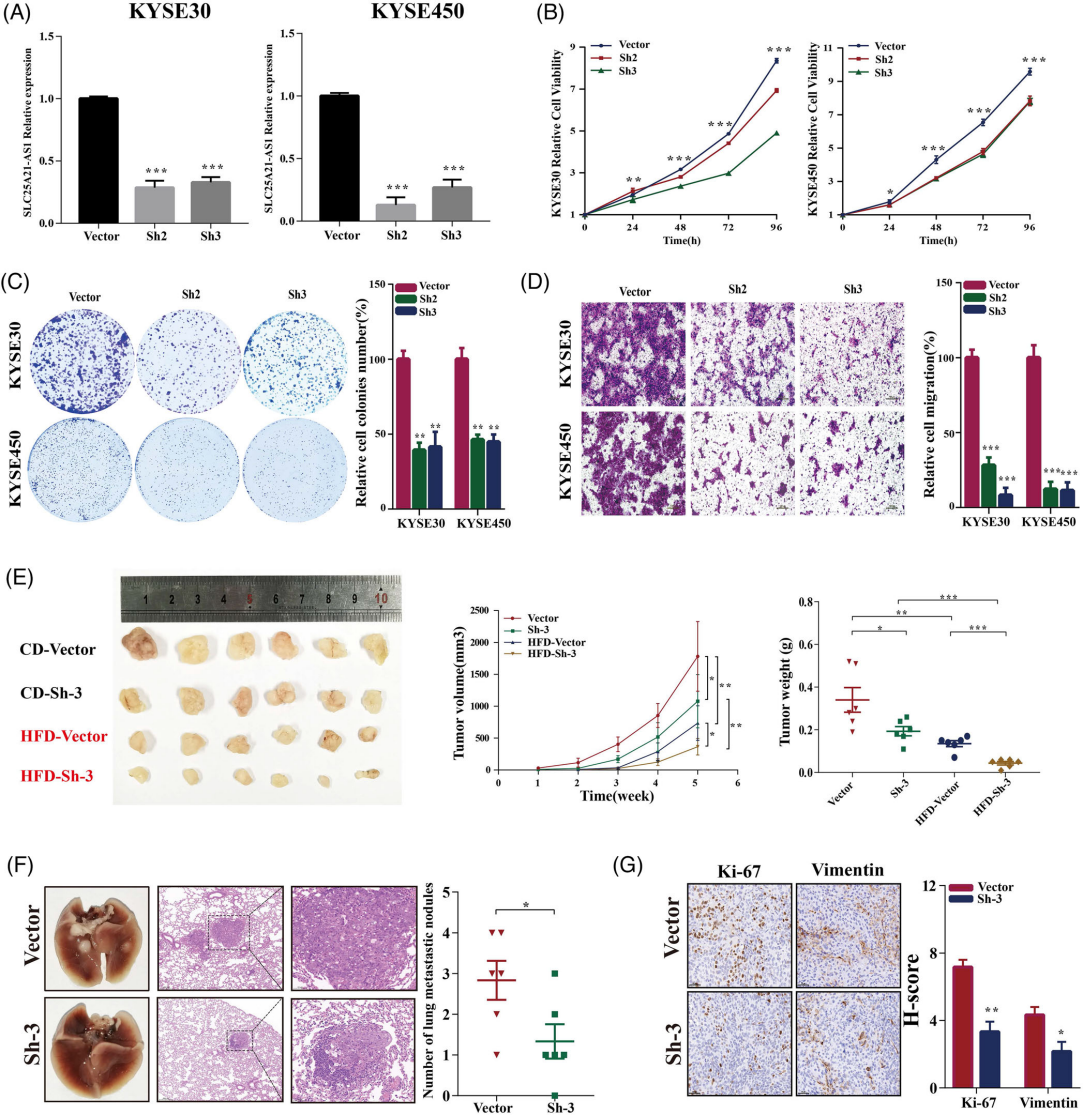
**Knockdown of *SLC25A21‐AS1* inhibits oesophageal squamous cell carcinoma (ESCC) proliferation and migration in vitro and in vivo**. (A) Validation of the *SLC25A21‐AS1* knockdown efficiency by RT‐qPCR in KYSE30 and KYSE450 cells. (B–D) The inhibitory effect of *SLC25A21‐AS1* knockdown on cell proliferation, colony formation, and migration. (E) The images, volumes, and weights of xenograft tumours established by the *SLC25A21‐AS1* knockdown and control KYSE30 cells; the inhibitory effect of *SLC25A21‐AS1* on tumour growth. The BALB/c nude mice were fed with a  control diet (CD) and a high‐fat diet (HFD), respectively. (F) Representative images of the gross lesion in lung tissues and haematoxylin and eosin staining of metastatic nodules in the lungs from the knockdown and vector groups. The right panel shows the quantification results of metastatic nodule numbers. (G) The left panel shows the representative images for Ki67 and vimentin immunostaining of tumour samples from the knockdown and vector groups. The right panel shows the quantification results of these immunochemistry assays. *p < .05; **p < .01; ***p < .001

Short hairpin RNAs (shRNAs) were used to knockdown *SLC25A21‐AS1* in KYSE30 and KYSE450 cells (Figure 4A), which resulted in the inhibition of cell proliferation, colony formation, and migration (Figure 4B–D). The data of the xenograft model showed that *SLC25A21‐AS1* knockdown decreased the tumour volumes in the CD group and significantly inhibited tumour growth in the HFD group (Figure 4E). The number of metastatic nodules in the *SLC25A21‐AS1* knockdown group was lower than the control group (Figure 4F). The data of IHC analysis showed that Ki67 and vimentin were expressed at low levels in the *SLC25A21‐AS1* knockdown group (Figure 4G).

### 2.4 *SLC25A21‐AS1* promotes tumour development through the NPM1/c‐MYC axis in the nucleus

**FIGURE 5:**
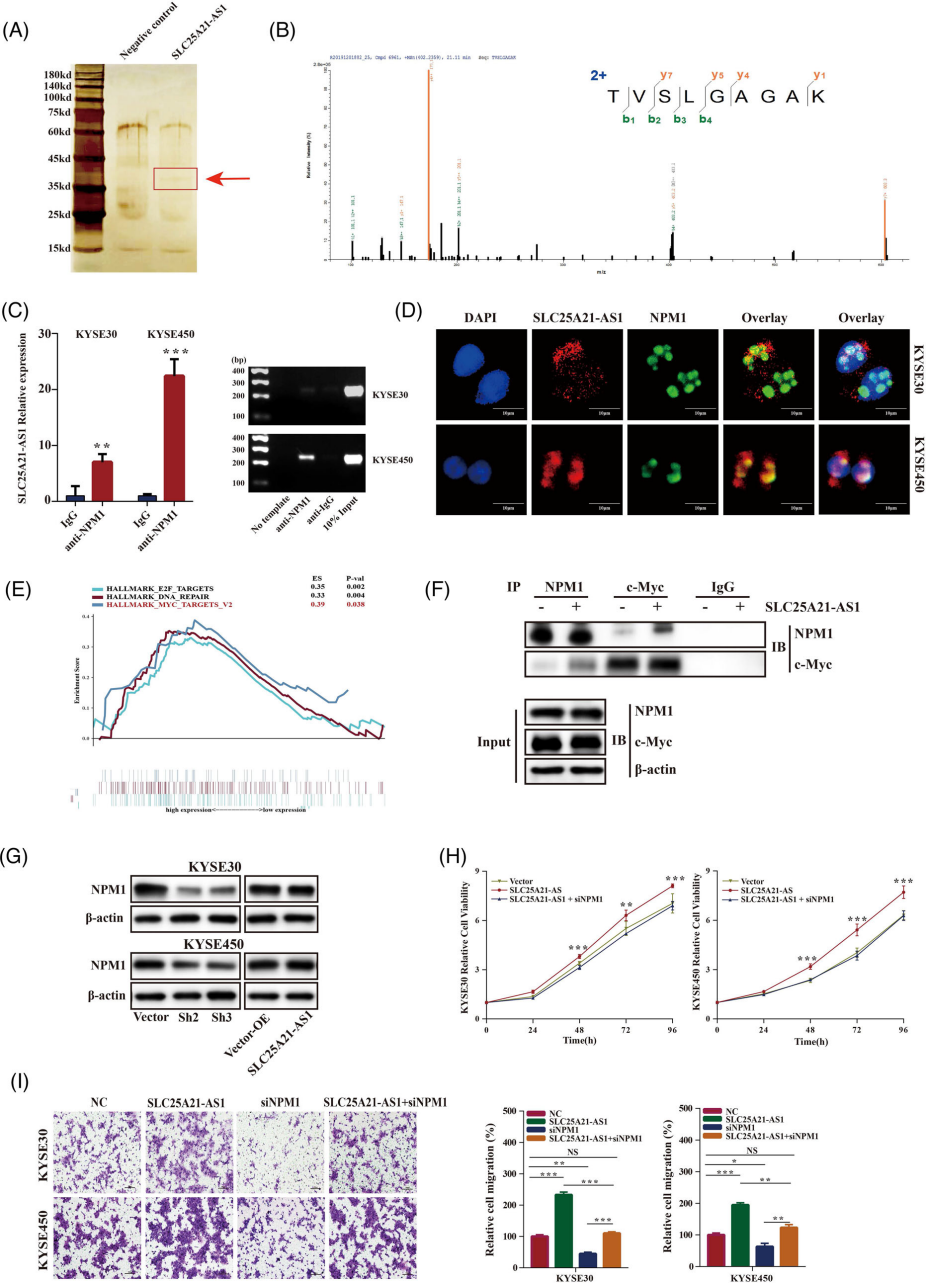
***SLC25A21‐AS1* promotes tumour development through interacting with nucleophosmin‐1 (NPM1) protein**. (A) Silver staining of *SLC25A21‐AS1*‐binding protein gel for the products detected by RNA‐pulldown. (B) A mass spectrometry picture for NPM1protein. (C) Validation of *SLC25A21‐AS1* binding to NPM1 protein by RT‐qPCR (left panel) and PCR gel electrophoresis (right panel). (D) the colocalization of *SLC25A21‐AS1* RNA (red) and NPM1 protein (green) in the nucleus as detected by RNA fluorescence in situ hybridization (FISH) and immunofluorescence staining. (E) Gene set enrichment analysis (GSEA) function enrichment analysis for the expression of *SLC25A21‐AS1* in the GSE52625 database. The Myc target pathway is indicated in the red font. (F) *SLC25A21‐AS1* overexpression enhanced the interaction between NPM1 and c‐Myc in KYSE30 cell lines, as detected by Co‐IP experiments. (G) NPM1 protein expression was downregulated in *SLC25A21‐AS1*‐knockdown oesophageal squamous cell carcinoma (ESCC) cells and upregulated in *SLC25A21‐AS1*‐overexpressing cell lines. (H) *SLC25A21‐AS1* overexpression promoted cell growth, which was abolished by NPM1 knockdown, as detected by the cell proliferation assay. (I) *SLC25A21‐AS1* overexpression promoted cell migration, which was inhibited or abolished by NPM1 knockdown, as detected by the Transwell assay. *p < .05, **p < .01 and ***p < .001

Several proteins that may interact with *SLC25A21‐AS1* were identified using the RNA pull‐down and mass spectrometry assays (Figures 5A,B and S4A). Nucleophosmin‐1 (NPM1) was verified as an SLC25A21‐AS1‐interacting protein using the RIP and RT‐qPCR assays in KYSE30 and KYSE450 cells (Figure 5C). Additionally, *SLC25A21‐AS1* and the NPM1 protein were colocalized in the nucleus, as demonstrated by RNA‐FISH and immunofluorescence staining (Figure 5D). NPM1 is a multifunctional nucleolar protein that plays an important role in tumour development. A previous study showed that the NPM1 protein could interact with Myc and enhance the transcription of the Myc target genes.^30^ In addition, the data of functional GSEA indicated a correlation between the expression of *SLC25A21‐AS1* and the Myc targets (Figures 5E and S4B). To explore possible function mechanisms, co‐IP was used to verify whether the NPM1 protein interacts with c‐Myc, and the results indicated that *SLC25A21‐AS1* overexpression enhanced the interaction between NPM1 and c‐Myc in KYSE30 cells (Figure 5F).

Next, we determined whether *SLC25A21‐AS1* regulates the NPM1/c‐Myc axis to influence the transcriptional activity of c‐Myc. The data of RT‐qPCR showed that the c‐Myc target genes, *EIF4E*, *NPL*, and *CDK4*, were upregulated in *SLC25A21‐AS1* overexpression cells (Figure S4C). NPM1 expression was regulated by *SLC25A21‐AS1* (Figure 5G). Hence, we knocked down the expression of NPM1 by siRNA, and the results indicated that the expression of three c‐Myc target genes was reduced (Figure S4D). Furthermore, the promoting effect of *SLC25A21‐AS1* on cell proliferation and migration was abolished when *NPM1* was knocked down (Figure 5H,I). In addition, NPM1 expression was decreased in the cells stimulated with PA and also associated with cell viability (Figure S4E,F). These results suggested that the promoting effect of *SLC25A21‐AS1* was mediated by the NPM1/c‐Myc axis in the nucleus.

### 2.5 *SLC25A21‐AS1* regulates the stability of *SLC25A21* mRNA in the cytoplasm

**FIGURE 6:**
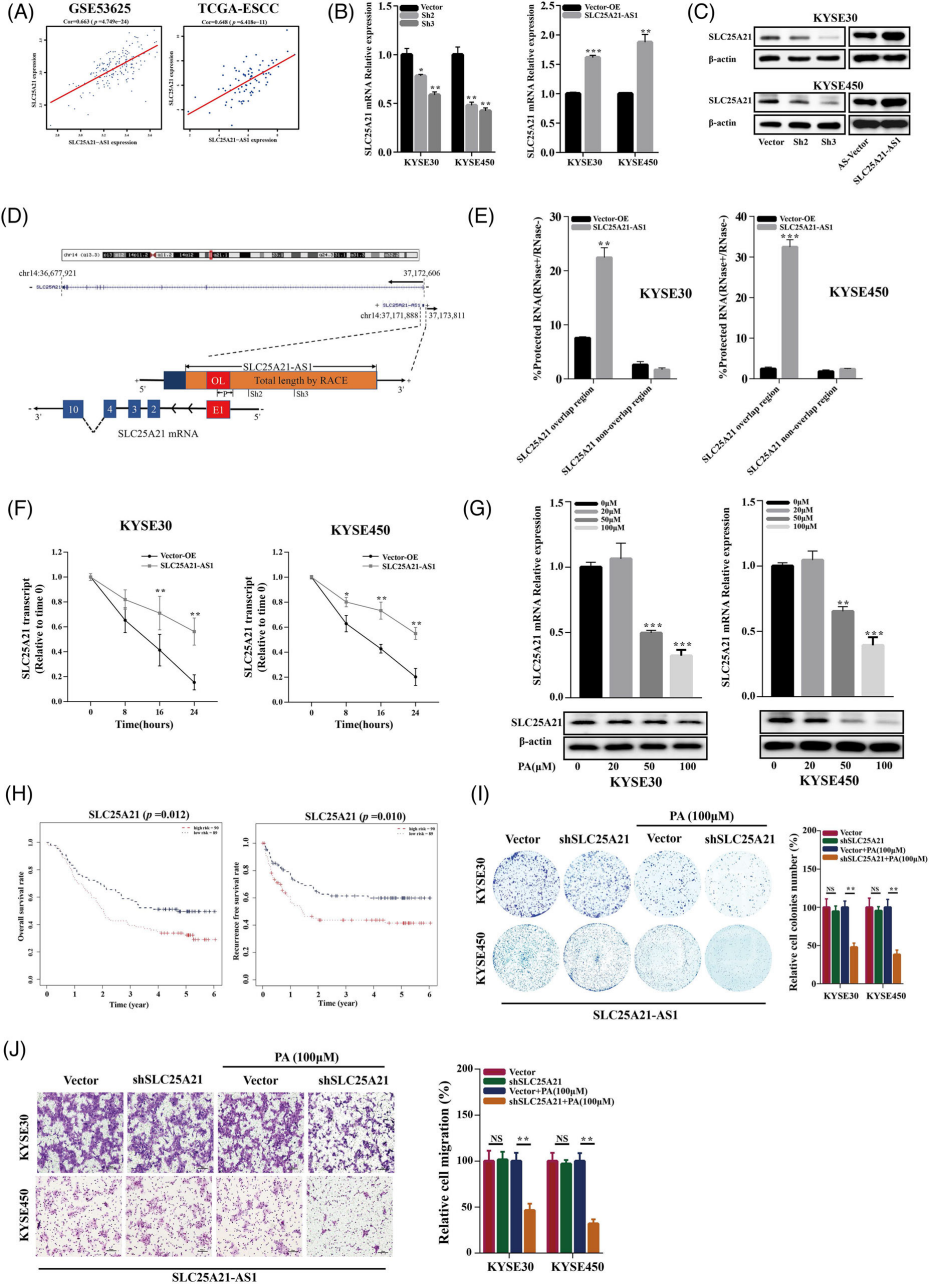
***SLC25A21‐AS1* regulates SLC25A21 expression by increasing the mRNA stability**. (A) The positive correlation between *SLC25A21‐AS1* and SLC25A21 mRNA expression in GSE53625 and TCGA datasets. (B) SLC25A21 mRNA expression was decreased in *SLC25A21‐AS1*‐knockdown oesophageal squamous cell carcinoma (ESCC) cells by RT‐qPCR. (C) SLC25A21 protein expression was decreased in *SLC25A21‐AS1*‐knockdown ESCC cells by Western blot. (D) Homology prediction analysis shows the start and end positions of *SLC25A21‐AS1* and SLC25A21 in the UCSC Genome Browser (the upper panel). The black arrows represent transcription directions, and the blue ones represent exons. “+” represents a positive strand, and “−” represents a negative strand. “P” represents the SLC25A21‐AS1 primer sequence. “Sh2” and “Sh3” represent the SLC25A21‐AS1 knockdown sequence. The schematic diagram for the overlapping (OL) region of *SLC25A21‐AS1* and SLC25A21 is shown in the lower panel. The orange region is the length of the *SLC25A21‐AS1* sequence as verified by the rapid amplification of cDNA ends (RACE) experiment, and the red OL is the overlapping region with the exon E1 of SLC25A21, which is also a possible complementary binding region. (E) RNase protection assay was performed on RNA samples from KYSE30 and KYSE450. The overlapping and non‐overlapping regions of *SLC25A21‐AS1* mRNA were detected in the overexpression *SLC25A21‐AS1* cell lines by RT‐qPCR. (F) The stability of SLC25A21 mRNA over time was detected by RT‐qPCR. *SLC25A21‐AS1*‐overexpressing ESCC cells were further exposed to 50 μg/mL α‐amanitin for 0, 8, 16, and 24 h. 18S RNA was used as an internal control. (G) The mRNA and protein expression of SLC25A21 in ESCC cells treated with different concentrations of palmitic acid (PA, 0–100 μM) as detected by RT‐qPCR and western blot, respectively. (H) The Kaplan–Meier survival curve of SLC25A21 expression for overall survival and recurrence‐free survival of ESCC patients in GSE53625 dataset. (I) Colony formation and (J) Transwell assays of cell proliferation in *SLC25A21‐AS1*‐overexpressing ESCC cells with or without 100‐μM PA when SLC25A21 was knocked down. The right panel shows the quantification results. *p < .05, **p < .01, and ***p < .001

An antisense lncRNA might regulate the expression of its cognate sense gene by forming the duplexes with cognate sense mRNAs to protect these mRNAs from ribonuclease degradation.^31^, ^32^, ^33^ We detected the coexpression of *SLC25A21‐AS1* and *SLC25A21* in the Gene Expression Omnibus (GEO) (GSE53625) and TCGA databases (Figure 6A) and verified this coexpression in ESCC cell lines (Figure 6B,C). The data of homology prediction analysis showed that *SLC25A21‐AS1* and *SLC25A21* mRNA may be able to form a protective duplex most likely at the complementary overlapping region (328 nucleotides) (Figure 6D). Then, an RNase protection assay was used to verify the presence of the overlapping and non‐overlapping regions in expressed mRNA using RT‐qPCR; the data indicated that the overlapping region was partially protected from RNase degradation in ESCC cells with *SLC25A21‐AS1* overexpression (Figure 6E). To further investigate whether the stability of SLC25A21 mRNA is regulated by *SLC25A21‐AS1*, ESCC cells were treated with α‐amanitin (50 μg/ml) for 8, 16, and 24 h; the data of RT‐qPCR showed that *SLC25A21‐AS1* overexpression significantly mitigated the degradation of *SLC25A21* mRNA (Figure 6F). These results indicate that *SLC25A21‐AS1* regulated the expression of *SLC25A21* by increasing the stability of *SLC25A21* mRNA.

*The SLC25A21* gene is a member of the mitochondrial carrier family (SLC25) that transports various metabolites, including 2‐oxoadipate and 2‐oxoglutarate by a counter‐exchange mechanism.^34^ The role of *SLC25A21* in tumours is unclear. We detected a decrease in *SLC25A21* expression after PA (100 μM) treatment (Figure 6G), and an increase in *SLC25A21* expression was associated with shorter OS and RFS according to the data of the GSE53625 dataset (Figure 6H). *SLC25A21* knockdown inhibited cell proliferation, reduced ATP levels, and increased ROS levels (Figure S5A–E). Moreover, the enhancing effect of *SLC25A21‐AS1* on the colony formation (Figure 6I) and migration (Figure 6J) achieved by 100‐μM PA treatment was mitigated or even abolished when *SLC25A21* was knocked down in *SLC25A21‐AS1* overexpressing cells.

**FIGURE 7:**
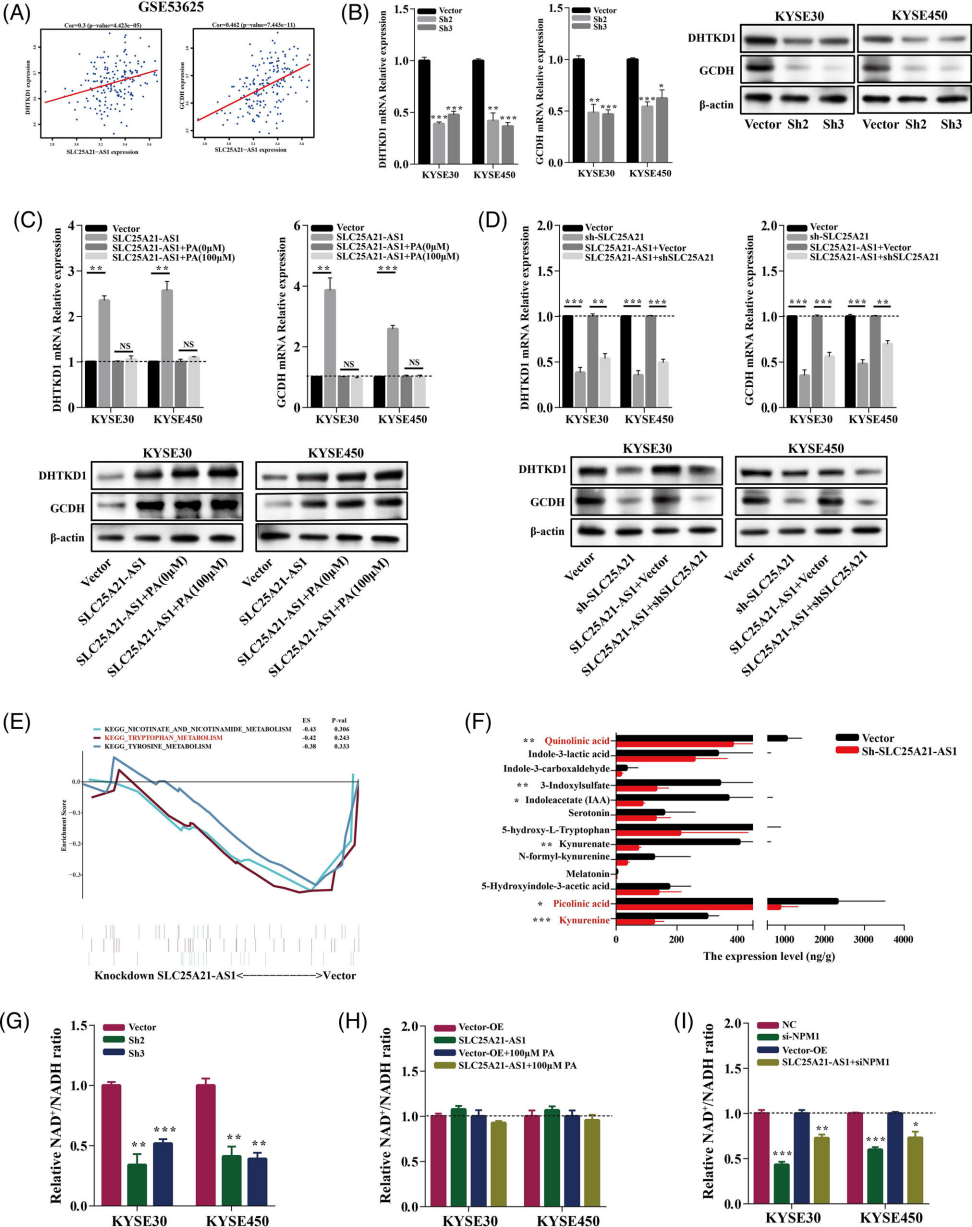
***SLC25A21‐AS1* affects intracellular tryptophan metabolism and NAD^+^ synthesis**. (A) The positive correlation between *SLC25A21‐AS1* and *DHTKD1* and *GCDH* mRNA expression in GSE53625 dataset. (B) mRNA and protein expression of DHTKD1 and GCDH were decreased in *SLC25A21‐AS1*‐knockdown cell lines. (C) mRNA and protein expression of DHTKD1 and GCDH was increased in *SLC25A21‐AS1*‐overexpresing cells with or without palmitic acid (PA) treatment. (D) mRNA and protein expression of DHTKD1 and GCDH were decreased when SLC25A21 expression was knocked down in *SLC25A21‐AS1*‐overexpressing cells lines. (E) The gene set enrichment analysis (GSEA) function enrichment analysis shows the downregulation of several KEGG pathways, including tryptophan metabolism (red font) in *SLC25A21‐AS1*‐knockdown cells as detected by RNA‐seq. (F) The tryptophan metabolites were decreased in *SLC25A21‐AS1*‐knockdown xenograft tumour tissues by targeted metabolomics. (G) The relative NAD^+^/NADH ratio was decreased in *SLC25A21‐AS1*‐knockdown oesophageal squamous cell carcinoma (ESCC) cell lines. (H) The relative NAD^+^/NADH ratio was not significantly changed in *SLC25A21‐AS1*‐overexpressing ESCC cells with and without PA treatment. (I) The relative NAD^+^/NADH ratio was significantly decreased in wild‐type and *SLC25A21‐AS1*‐overexpressing ESCC cells with nucleophosmin‐1 (NPM1)‐knockdown. *p < .05, **p < .01, and ***p < .001

A previous study showed that *SLC25A21* deficiency is associated with the levels of several metabolites such as 2‐oxoadipate and quinolinic acid.^35^ 2‐Oxoadipate is a common intermediate in the catabolism of tryptophan, hydroxylysine, and lysine and is one of the molecules transported by *SLC25A21*. 2‐Oxoadipate can be converted into acetyl‐CoA in the mitochondria to participate in the tricarboxylic acid (TCA) cycle.^34^ The proteins encoded by *DHTKD1* and *GCDH* genes are involved in the catabolic pathways of lysine, tryptophan, and hydroxylysine after *SLC25A21* transports the metabolites such as 2‐oxoadipate into the mitochondria.^36^, ^37^ We demonstrated that *SLC25A21‐AS1* expression was correlated with the expression of *DHTDK1* and *GCDH* (Figure 7A), implying an association between *SLC25A21‐AS1* and the catabolism of amino acids. The expression of DHTKD1 and GCDH mRNAs and proteins was decreased in *SLC25A21‐AS1*‐knockdown cells (Figure 7B) and increased in *SLC25A21‐AS1*‐overexpressing cells; moreover, the expression was not influenced by the treatment with 100‐μM PA (Figure 7C). In addition, *SLC25A21* knockdown in *SLC25A21‐AS1*‐overexpressing cells significantly downregulated the expression of *DHTKD1* and *GCDH* (Figure 7D). These results indicated that *SLC25A21‐AS1* may influence amino acid catabolism by regulating the expression of SLC25A21.

### 2.6 *SLC25A21‐AS1* knockdown has an inhibitory effect on tryptophan metabolism

The data of GSEA showed that several metabolic pathways were enriched by *SLC25A21‐AS1* knockdown (Figure 7E). Tryptophan (Trp) is an essential amino acid, and the kynurenine (Kyn) pathway is the principal route of catabolism associated with de novo synthesis of NAD^+^ and with several biologically active metabolites.^38^ Previous studies have shown that Myc promotes tryptophan uptake and activates the Kyn pathway.^39^ We explored the effect of *SLC25A21‐AS1* on tryptophan catabolism by targeted metabolomic analysis in the xenograft tumour tissue. The data indicated that the levels of several metabolites, including kynurenine and picolinic acid, were reduced, and that the NAD^+^/NADH ratio was decreased when *SLC25A21‐AS1* was knocked down (Figure 7F,G). In addition, *SLC25A21‐AS1* overexpression did not increase the NAD^+^/NADH ratio and reversed a decrease in the NAD^+^/NADH ratio induced by 100‐μM PA (Figure 7H). However, NPM1 knockdown significantly decreased the NAD^+^/NADH ratio in *SLC25A21‐AS1*‐overexpressing cells (Figure 7I). These results indicated that *SLC25A21‐AS1* influenced the NAD^+^/NADH ratio by regulating tryptophan metabolism.

### 2.7 *SLC25A21‐AS1* may serve as a biomarker of favourable prognosis and therapeutic target

**FIGURE 8:**
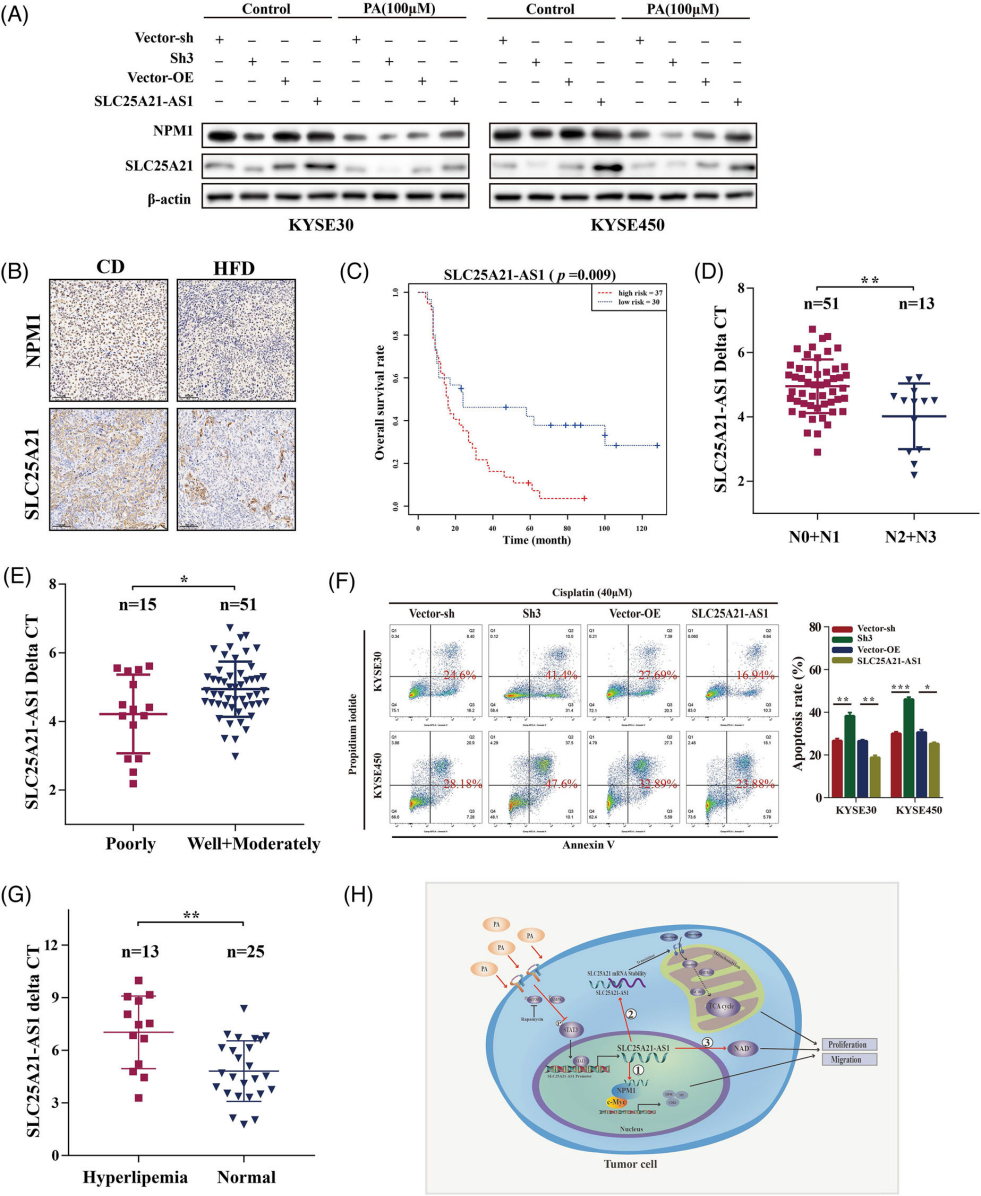
**The favourable prognosis biomarker and potential therapeutic target of *SLC25A21‐AS1* for oesophageal squamous cell carcinoma (ESCC) patients**. (A) Nucleophosmin‐1 (NPM1) and SLC25A21 protein expression in *SLC25A21‐AS1*‐knockdown and overexpressing ESCC cells were treated with palmitic acid (PA) (100 μM). (B) NPM1 and SLC25A21 protein expressions were lower in the high‐fat diet (HFD) group (*n* = 6) than in the control diet (CD) group (*n* = 6) as detected by immunohistochemical analysis. (C) The Kaplan–Meier survival curve showed that *SLC25A21‐AS1* expression was associated with overall survival in 67 ESCC patients as determined by tissue cDNA microarrays by RT‐qPCR: (D and E) Significant association between *SLC25A21‐AS1* expression and some clinical characteristics including N stage and tumour grade differentiation as determined by tissue cDNA microarrays. (F) Apoptosis analysis showed that under treatment with 40‐μM cisplatin, apoptosis rate was significantly increased in *SLC25A21‐AS1*‐knockdown ESCC cells but decreased in *SLC25A21‐AS1*‐overexpressing cells. (G) The expression of *SLC25A21‐AS1* was low expression in tumour tissues of 38 ESCC patients with hyperlipidaemia (*n* = 13) than without hyperlipidaemia (*n* = 25) by RT‐qPCR. (H) The graphical summary of the function and mechanism of *SLC25A21‐AS1*. *p < .05, **p < .01, and ***p < .001

We demonstrated that *NPM1* and *SLC25A21* expression was inhibited by PA and *SLC25A21‐AS1* knockdown, and *SLC25A21‐AS1* overexpression enhanced the expression of these two genes and reversed the inhibitory effect of PA (Figure 8A). The data of IHC analysis showed that NPM1 and *SLC25A21* expression was decreased in the HFD group (Figures 8B and S5F). The results of public database analysis indicated that the expression of *SLC25A21‐AS1* was different in different tumours (Figure S6A). Moreover, low SLC25A21‐AS1 expression in the ESCC tumour tissues was indicated by the data of the TCGA and GTEx datasets (Figure S6B); however, we did not detect significant differences between 179 paired tumours and adjacent normal tissues samples of the GSE53625 dataset or in 28 paired tissues samples of the cDNA microarray (Figure S6C,D). The data of subsequent analysis showed that high *SLC25A21‐AS1* expression was associated with poor prognosis, late N stage, and poor tumour grade in 67 ESCC patients tested using cDNA microarrays (Figure 8C–E). The detailed information is shown in Table 1. Moreover, *SLC25A21‐AS1* expression was decreased in patients with hyperlipidaemia in an independent validation group of 38 ESCC patients and was associated with OS (Figures 8G and S6E; Table S4). These results indicated that *SLC25A21‐AS1* expression may be a prognostic biomarker for ESCC patients.

**TABLE 1:** The clinicopathological characteristics stratified by prognostic related group in 67 oesophageal squamous cell carcinoma (ESCC) patients

	SLC25A21‐AS1 expression (delta *Ct*)			
Variables	Low group	High group	Total	*p*‐Value
Overall survival	42.267 ± 38.078	23.838 ± 19.874	67	.013*
Age				.585
<60	19 (63.33%)	21 (56.76%)	40	
≥60	11 (36.67)	16 (43.24%)	27	
Gender				.788
Female	9 (30.00%)	10 (27.03%)	19	
Male	21 (70.00%)	27 (72.97%)	48	
Tumour grade				.599
Well	3 (10.00%)	2 (5.41%)	5	
Moderately	20 (66.67%)	26 (70.27%)	46	
Poorly	6 (20.00%)	9 (24.32%)	15	
T stage				.869
T1	1 (3.33%)	1 (2.30%)	2	
T2	5 (16.67%)	8 (21.62%)	13	
T3	16 (53.33%)	22 (59.46%)	38	
T4	1 (3.33%)	1 (2.70%)	2	
N stage				.231
N0	15 (50.00%)	18 (48.65%)	33	
N1	10 (33.33%)	8 (21.62%)	18	
N2	1 (3.33)	8 (21.62%)	9	
N3	2 (6.67%)	2 (6.41%)	4	
M stage				.477
M0	27 (90.00%)	35 (94.59%)	62	
M1	3 (10.00%)	2 (5.41%)	5	
TNM stage				.779
Ι	1 (4.54%)	1 (3.03%)	2	
II	9 (40.91%)	16 (48.48%)	25	
III	9 (40.91%)	14 (42.42%)	23	
Death at FU				.001***
No	11 (36.67%)	2 (5.41%)	13	
Yes	19 (63.33%)	35 (94.59%)	54	

*p < .05; **p < .01; ***p < .001.

In addition, a previous study showed that *SLC25A21‐AS1* is associated with multidrug resistance.^27^ The present study demonstrated that the rate of cisplatin‐induced apoptosis was significantly increased in *SLC25A21‐AS1*‐knockdown cells and decreased in SLC25A21‐AS1‐overexpressing cells (Figure 8F). These results indicated that *SLC25A21‐AS1* may be associated with drug resistance and is a potential therapeutic target.

## 3 DISCUSSION

Several factors, such as socioeconomic status, environment, diet, BMI, microbiome, and genetic factors, contribute to the occurrence and development of ESCC.^40^ HFD is the main dietary factor leading to obesity, and fatty acids in HFD may serve as the energy source for tumour cells and are associated with the development of tumours, such as prostate cancer and breast cancer.^16^, ^41^, ^42^, ^43^, ^44^, ^45^ However, HFD inhibits the peritoneal seeding of colorectal cancer through activation of ATMs and enhancement of tumour phagocytosis by ATMs.^15^ The relationship between HFD and ESCC has not been explored. The present study established an animal model of HFD‐induced obesity to demonstrate that HFD increased the weight and blood lipid levels in mice. These findings are consistent with the data obtained in a previous study.^43^

The levels of several fatty acids, especially PA, in tumour tissues were significantly different between the HFD and CD groups. PA is a long‐chain saturated fatty acid that acts as a signalling molecule to influence tumour development.^16^ A previous study used nine types of lung cancer cells to identify the two lipid‐sensitive A549 and H1688 cell lines, which were significantly inhibited by various doses of PA.^46^ The heterogeneity of the ESCC cell lines should be considered. The fatty acid receptor CD36 on the surface of the cells is responsible for lipids uptakes from the extracellular microenvironment to promote fatty acid oxidation, potentially activating tumour progression and metastasis.^47^, ^48^ The tumour‐promoting effect of PA may be mediated by CD36.^49^ PA also improves the metastatic ability of CD36+ metastasis‐initiating oral cancer cells and promotes proliferation and invasiveness of melanoma.^50^, ^51^, ^52^ A previous study demonstrated that only 18.8% of ESCC tumour tissues express high levels of CD36 according to the data of IHC staining, and the expression of CD36 is not detected in several ESCC cell lines.^53^ We also demonstrated that CD36 was expressed at low levels according to the data of the GSE53625 dataset and had variable expression levels in the ESCC cell lines (Figure S7A,B). Hence, we determined whether CD36 expression is associated with the effect of HFD on ESCC by selecting the other three ESCC cell lines according to the level of CD36 expression. Our study demonstrated a significant inhibitory effect of HFD on KYSE450 cells with low CD36 expression and a trend towards inhibition in KYSE70 and KYSE150 cells with relatively high CD36 expression (Figure S7C–H). These results indicated that the heterogeneity of tumour cells and local metabolic differences may be partly related to the function of HFD/PA. A previous study showed an inhibitory effect of PA on cell proliferation, invasiveness, and tumour growth by affecting the membrane parameters, dysregulating energy metabolism, and influencing mTOR pathway phosphorylation in hepatocellular cancer.^54^ The present study demonstrated an inhibitory effect of PA only on ESCC cells but not on Het1A cells (Figure S1D). In addition, PA inhibited the phosphorylation of mTOR and STAT3 and activated the phosphorylation of AMPK (Figure S8A–C). The inhibitory effect of PA on *SLC25A21‐AS1* expression was regulated via the mTOR/STAT3 pathway in ESCC cells (Figure S8D). We also confirmed that STAT3 was able to bind the *SLC25A21‐AS1* promoter and influenced the transcription of *SLC25A21‐AS1* (Table S5; Figure S8E–H). The AMPK signalling pathway is associated with cellular energy homeostasis, and the mTOR signalling pathway is closely associated with tumourigenesis, especially with the changes in tumour metabolism.^55^, ^56^ These findings indicate that exogenous PA may interfere with energy homeostasis in ESCC.

Previous studies have demonstrated that NPM1 interacts with several nuclear proteins such as HDM2 and Myc.^30^, ^57^ NPM1 interacts with Myc to form the NPM1–Myc binary complex, which induces the transcription of the Myc target genes and promotes Myc‐induced hyperproliferation and transformation.^30^ Our study indicated that *SLC25A21‐AS1* interacted with the NPM1 protein and enhanced the binding of NPM1 to c‐Myc and the transcription and expression of the downstream target genes regulated by Myc (Figure S4C). Myc overexpression has been shown to induce global metabolic reprogramming to support cancer cell survival and growth.^58^, ^59^ Myc increases tryptophan uptake and activates the Kyn pathway in tumour cells, promoting the catabolism in the tryptophan metabolic pathway.^39^ We demonstrated that *SLC25A21‐AS1* was related to multiple metabolic pathways, including the tryptophan metabolic pathway (Figure 7E). In addition, the levels of the intermediates of tryptophan metabolism, including quinolinic acid, were significantly reduced in the ESCC tissues (Figure 7F). Quinolinic acid is one of the degradation products of tryptophan catabolism and can be used as a precursor participating in the synthesis of NAD^+^.^38^, ^60^, ^61^ NAD^+^ is a fundamental signalling cofactor that regulates tumour metabolism, including glycolysis, the TCA cycle, and oxidative phosphorylation.^62^ In the present study, the downregulated *SLC25A21‐AS1* expression was associated with tryptophan metabolism and a low NAD^+^/NADH ratio, possibly reflecting the metabolic alterations. Thus, these findings indicate that *SLC25A21‐AS1* influenced tumour metabolism and progression by regulating the NPM1/c‐Myc axis.

Antisense lncRNAs bind to mRNA and protect it from the degradation regulated by RNases or microRNAs.^31^, ^32^, ^33^ *SLC25A21‐AS1* regulated the expression of *SLC25A21* by increasing the stability of *SLC25A21* mRNA in the cytoplasm, and downregulation of *SLC25A21* was closely associated with the proliferation of ESCC cells. *SLC25A21* is a mitochondrial 2‐oxodicarboxylate carrier, and abnormal transport of 2‐oxodicarboxylate disrupts the pathway of lysine and tryptophan degradation, leading to the accumulation of 2‐oxoadipate and quinolinic acid.^35^ 2‐Oxoadipate can be converted into acetyl‐CoA in the mitochondria by DHTKD1 and GCDH.^34^, ^35^, ^36^, ^37^ Considering that the levels of quinolinic acid and SLC25A21 were decreased when *SLC25A21‐AS1* was knocked down, we speculated that the downregulation of *SLC25A21* expression not only affected cell proliferation but also played a compensatory role to partially prevent a subsequent decline in quinolinic acid and to promote the synthesis of NAD^+^ (Figure 8H). The possible mechanism of these effects needs additional studies. Overall, these findings indicated that *SLC25A21‐AS1* maintained the stability of *SLC25A21* mRNA to enhance its expression in the cytoplasm to regulate tumour metabolism.

Previous studies have shown that several lncRNAs are potential prognostic biomarkers or are associated with chemosensitivity in ESCC.^63^, ^64^, ^65^, ^66^ The present study initially confirmed that *SLC25A21‐AS1* expression was significantly correlated with tumour differentiation and OS. Cisplatin is a standard treatment for ESCC, and chemoresistance to this drug remains a major problem.^67^, ^68^ A previous study indicated that *SLC25A21‐AS1* is associated with multidrug resistance.^27^ The present study demonstrated that *SLC25A21‐AS1* overexpression conferred resistance to cisplatin‐induced apoptosis, whereas *SLC25A21‐AS1* knockdown enhanced chemosensitivity. Currently, antisense oligonucleotides, RNA interference (RNAi) technology, small molecule inhibitors, and CRISPR/Cas9 are therapeutic strategies targeting lncRNAs.^69^ The downregulation of SLC25A21‐AS1 expression inhibited tumour progression and enhanced chemosensitivity in the present study. These results indicated the therapeutic potential of SLC25A21‐AS1 in ESCC.

There are a couple of limitations in the present study. First, immunodeficient mice used in the present study were unable to manifest the comprehensive effect of the tumour microenvironment on tumour growth. Therefore, immune‐related factors should be considered in future studies. Second, the influence of obesity on the tumour is very complex, and HFD is only one factor predisposing for obesity; hence, the results of animal experiments are expected to be different from the actual situation. Discrepancies with other studies may be due to the differences in metabolic reprogramming in different types of cancers.^16^ For example, CD36 expression or the levels of related lipogenic enzymes are different in various types of cancer cells. The present study was unable to detect the comprehensive effect of PA on the tumour microenvironment in the context of tumour growth. Therefore, the effect of immune‐related factors and stromal cells should be considered in future studies. Finally, HFD contained several fatty acids, and the present study investigated only a single important fatty acid (i.e., PA). The effects of other fatty acids on tumour growth need further exploration.

In conclusion, HFD/PA has an inhibitory effect on ESCC cells and *SLC25A21‐AS1* expression. *SLC25A21‐AS1* promoted the proliferation and migration of ESCC cells through the NPM1/c‐Myc axis and an increase in *SLC25A21* expression. LncRNA *SLC25A21‐AS1* was associated with OS, tumour grade, and cell apoptosis, indicating that lncRNA *SLC25A21‐AS1* may serve as a favourable prognostic biomarker and may be a potential therapeutic target for ESCC.

## 4 MATERIALS AND METHODS

### 4.1 Patients and tissue samples

Thirty‐eight samples of ESCC patient tissues were collected retrospectively; these patients underwent surgical treatment at our hospital from January 2012 to December 2012, and patients did not receive neoadjuvant chemoradiotherapy before surgery. An ESCC cDNA microarray included 28 paired samples of the tumour and adjacent tissues and 39 individual samples of the tumour tissues obtained from OUTDO BIOTECH (HEsoS095Su01, Shanghai). Total RNA was isolated, and RT‐qPCR was performed to evaluate the expression of *SLC25A21‐AS1* and assess the correlations with the prognosis and clinicopathological characteristics of patients. The delta *Ct* method was used to evaluate gene expression based on the data normalized to the levels of β‐actin.

### 4.2 Cell culture

The ESCC KYSE30, KYSE450, KYSE150, KYSE70, KYSE140, KYSE510, and KYSE180 cell lines were cultured in RPMI‐1640 medium (HyClone) with 10% FBS (Gibco). Het‐1A oesophageal epithelial cells were cultured in BEGM (Lonza/Clonetics). The cell lines were incubated at 37°C and 5% CO_2_.

PA (#P9767, Sigma‐Aldrich), stearic acid (#S4751, Sigma‐Aldrich), and oleic acid (#O1008, Sigma‐Aldrich) were dissolved in ddH_2_O at 70°C, filtered through a .4‐μm filter and stored at −20°C. The stock solutions were added to a 2% fatty acid‐free bovine serum albumin (BSA, #A7030, Sigma‐Aldrich) medium made by dissolving FA‐free BSA in serum‐free RPMI‐1640 medium. Rapamycin (HY‐10219, MCE) was stored as 10‐mM stock solution in DMSO.

### 4.3 RNA isolation and RT‐qPCR

Total RNA was isolated using the TRIzol protocol (Thermo). Cytoplasmic and nuclear RNAs were extracted and purified using a PARIS kit (AM1921, Thermo). cDNA synthesis was performed using EasyScript All‐in‐one First step cDNA Synthesis SuperMix for qPCR (AE341, TransGen). qPCR was performed using PerfectStart Green qPCR SuperMix (AQ602, TransGen) and an ABI 7900HT real‐time PCR thermocycler (Life Technologies). The gene expression data were normalized to the results of the endogenous control β‐actin. GAPDH was used as a cytoplasmic control, and U99 was used as a nuclear control. The 2^−ΔΔ^ *^Ct^* method was used, and all samples were analysed in triplicate. The primer sequences are shown in Table S1.

### 4.4 Western blotting and co‐immunoprecipitation (co‐IP)

Total proteins were used and concentration was quantified with a BCA protein assay kit (#23227, Thermo). The proteins were separated by SDS‐PAGE and transferred to PVDF membranes (Millipore). The membranes were blocked with 5% fat‐free milk or 5% BSA and immunoblotted with appropriate antibodies. We used the Pierce Classic Magnetic IP/Co‐IP Kit (#88804, Thermo) to perform the co‐IP experiment.

Antibodies for western blotting, against AMPKα (#5831), p‐AMPKα (#2537), mTOR (#9964), p‐mTOR (#9964), STAT3 (#9139), p‐STAT3 (#9134), and β‐actin (#4970) were purchased from Cell Signaling Technology (CST). Antibodies against SLC25A21 (ab167033), NPM1 (ab10530, ab208015), and c‐Myc (ab32072) were purchased from Abcam. Antibodies specific to DHTKD1(A8369) and GCDH(A9057) were purchased from ABclonal. CD36(18836‐1) was purchased from Proteintech.

### 4.5 The acquisition of full‐length cDNA sequences by RACE

The SMARTer RACE 5′/3′ Kit (634923, TaKaRa) was used to obtain the 3′ RACE and 5′ RACE fragments according to the manufacturers’ instructions. The 3′ RACE and 5′ RACE PCR products were cloned into the pEASY‐Blunt Zero vector using a pEASY‐Blunt Zero Cloning Kit (CB501, TransGen).

### 4.6 Plasmid transfection and RNA interference

shRNAs were cloned into a pLV‐hU6‐puro vector and used to knock down the non‐overlapping region of SLC25A21‐AS1 (SyngenTech, China). Full‐length SLC25A21‐AS1 cDNA was inserted into a GV367 vector system (GeneChem, China) for the overexpression of *SLC25A21‐AS1*. The shRNA of the non‐overlapping region of *SLC25A21* was cloned into the phU6‐MCS‐neom vector (GeneChem, China). The packaging plasmids were transfected into HEK‐293T cells with Lipofectamine 3000 (L3000075, Thermo) to obtain infectious lentivirus particles. The corresponding empty vectors were used as controls. Puromycin (P9620, Sigma‐Aldrich) and G418 (10131035, Thermo) were used to screen stable cell lines after 1–2 weeks.

Small interfering RNAs (siRNAs) were designed to knock down the *NPM1* expression. The siRNAs were synthesized by SyngenTech in China. siRNA (50 pmol) and RNAiMAX (13778500, Thermo) were used. The shRNA and siRNA sequences are shown in Table S2.

### 4.7 Chromatin immunoprecipitation (ChIP) assay

We used the Simple ChIP Enzymatic Chromatin IP Kit (#9005, CST). The ESCC cell lines were treated with 0‐ or 100‐μM PA for 24 h prior to ChIP assays. The sequence ranging from 2000‐bp upstream to 100‐bp downstream was considered the promoter region. The possible areas of convergence were predicted through the JASPER database, and specific primers were designed.

### 4.8 RNA pull‐down, mass spectrometry, and RIP assays

Initially, the linearized vector Pet28a_T7 promoter‐*SLC25A21‐AS1* containing full‐length *SLC25A21‐AS1* cDNA was generated and transcribed in vitro using a HiScribe T7 High Yield RNA Synthesis Kit (E2040S, NEB). Then, we used a Pierce Magnetic RNA‐Protein Pull‐Down Kit (20164, Thermo) to label SLC25A21‐AS1 and the negative RNA control poly(A)25 RNA with biotin for subsequently captured on streptavidin magnetic beads. Finally, the RNA‐binding protein complexes of SLC25A21‐AS1 and a negative RNA control were subjected to SDS gel electrophoresis and silver staining (Sangon Biotech, China), and specific bands were selected for mass spectrometry detection (APTBIO, China).

In addition, we used a Magna RIP RNA‐Binding Protein IP Kit (17‐700, Millipore). The ESCC cell lines were lysed in complete RIP lysis buffer and incubated with magnetic beads conjugated with specific antibodies or IgG for at least 12 h at 4°C. The purified RNAs were used for cDNA synthesis and RT‐qPCR.

### 4.9 Dual luciferase reporter assay

The SLC25A21‐AS1 promoter and mutant vector were cloned into the pGL3‐basic vector and synthesized (Generay Biotech, China). The STAT3 cDNA was cloned into the pcDNA3.1(+) vector. The luciferase activities were detected using a Dual‐Luciferase Reporter Assay System (Promega).

### 4.10 HFD‐induced animal model of obesity and animal experiments in vivo

Four‐week‐old male athymic BALB/cA‐nu mice (Huafukang Bioscience, China) were fed a CD (13.8% fat energy) or an HFD (60% fat energy) for 12 weeks and subcutaneously injected with 1 × 10^6^ KYSE30 cells into their right flanks for 4–5 weeks. Body weight was determined once weekly, and plasma samples were collected after feeding for 12 weeks. The tumour size was determined once a week. After 4–5 weeks, we obtained the tumour tissues, calculated the tumour volume using the equation *V* = length × width^2^/2, and estimated the tumour weight.

BALB/cA‐nu mice were treated with PA (10.26 mg/kg/day) by gavage as described in a previous study 1 week after the injection of the KYSE30 cell line.^54^ The negative control group received 2% fatty acid‐free BSA daily. In addition, cell lines with stable knockdown or overexpression of *SLC25A21‐AS1* and the cells treated with the corresponding vector controls were injected into CD‐ and HFD‐fed mice. NOD‐SCID mice were used to study tumour metastasis 8–12 weeks after the cells were injected in the tail vein.

### 4.11 Cell proliferation, apoptosis, Transwell, ATP, ROS, and NAD^+^/NADH assays

Cell Counting Kit‐8 (CCK‐8) assays (KGA317, KeyGEN) were used to detect cell proliferation. To measure the IC50 values, KYSE30 (3000 cells/well) and KYSE450 (2500 cells/well) cells were stimulated with 0, 5, 10, 20, 50, 75, 100, 150, or 200 μM PA for 24 h, and the OD values were determined. Apoptosis was evaluated by using an Annexin V‐FITC/APC and propidium iodide (PI) apoptosis detection kit (KGA108/KGA1030, KeyGEN).

The Transwell chambers (#3422, Corning) were used for the migration assays. Briefly, cells were serum‐starved for 24 h, trypsinized, and resuspended in a serum‐free medium. A total of 5 × 10^5^ KYSE30 and 15 × 10^5^ KYSE450 cells were seeded into a Transwell chamber. At least five fields of views were analysed by light microscopy (100×), and the number of cells was counted. An NAD^+^/NADH assay kit (S0179, Beyotime), an ATP assay kit (S0027, Beyotime), and an ROS assay kit (S0033S, Beyotime) were used.

### 4.12 RNA‐FISH and immunofluorescence assay

RNA‐FISH was performed using an *SLC25A21‐AS1*‐specific probe (Servicebio, China). The probe sequence was 5′‐CY3‐TTCTGATTCCGTTTAGGTCGGGGTGG‐CY3‐3′. Cells were fixed with paraformaldehyde for 20 min and incubated with the *SLC25A21‐AS1* probe overnight at 37°C, followed by washing and blocking with 3% BSA. Subsequently, the cells were incubated with an NPM1 antibody for 2 h. The cells were incubated with an Alexa Fluor 488–conjugated secondary anti‐mouse antibody (#4408, CST). The cell nuclei were stained with DAPI.

### 4.13 RNA sequencing

Briefly, 100‐μM PA‐treated and control cells, *SLC25A21‐AS1*‐knockdown and control cells were selected for RNA‐seq. Illumina PE150 was used for RNA‐seq which was carried out by Novogene (China).

### 4.14 Immunohistochemical staining (IHC)

Anti‐Ki67(#9449), anti‐vimentin (#5741), anti‐NPM1 (ab10530), and anti‐SLC25A21(ab167033) antibodies were used for IHC of subcutaneous xenograft tumour tissue. The lung tissue was stained with haematoxylin and eosin. The staining score was calculated as intensity score × percentage score.

### 4.15 Metabolomic analysis

Medium‐ and long‐chain fatty acids were analysed by GC–MS‐based targeted metabolomics of xenograft tumour tissues (APTBIO, China). Briefly, a standard curve was constructed by NU‐CHEK‐PREP using 37 standards of fatty acid methyl ester mixtures; extraction of metabolites and mass spectrometry were performed by an Agilent 7890A/5975C GC–MS system (Agilent, US). Finally, MSD ChemStation software was used to calculate the fatty acid content.

The detection of tryptophan metabolites was performed based on the MRM method for xenograft tumour tissues (APTBIO, China) using a Sciex 5500 QTRAP mass spectrometer. MultiQuant and Analyst software were used to calculate the contents of the metabolites.

### 4.16 Ribonuclease protection assay (RPA)

We designed the primers specific to the overlapping and non‐overlapping regions of *SLC25A21*. The primer sequences are listed in Table S1. The RNA samples from two *SLC25A21‐AS1*‐overexpressing cell lines, KYSE30, and KYSE450, were incubated at 37°C for 1 h before treatment with an RNase A + T cocktail (AM2286, Thermo). After adding the RNase A + T cocktail, the RNA samples were incubated for 30 min at 37°C. The RNA samples were treated with proteinase K for 1 h at 50°C. Then, RNA was purified from the samples. RT‐qPCR was used to quantify the expression of overlapping and non‐overlapping regions of *SLC25A21*.

### 4.17 mRNA stability analysis

*SLC25A21‐AS1* was overexpressed in KYSE30 and KYSE450 cells. The ESCC cell lines were treated with α‐Amanitin (HY‐19610, MCE), an inhibitor of RNA polymerase II that blocks de novo RNA synthesis. RNA was harvested for RT‐qPCR 8, 16, and 24 h after the addition of α‐amanitin (50 μg/ml). 18S ribosomal RNA is synthesized by RNA polymerase I and is not affected by α‐amanitin treatment. Hence, 18S ribosomal RNA was used as an internal control. Three independent experiments were performed.

### 4.18 Bioinformatics analysis

The GSE53625 dataset was obtained from GEO, which contained the data of a tumour lncRNA + mRNA microarray of 179 ESCC patients and the clinical information.^19^, ^70^ Kaplan–Meier survival curves and log‐rank tests were used to calculate the differences in prognostic parameters between the groups. The data on the tumour tissues of 80 ESCC patients were obtained from TCGA and Genotype‐Tissue Expression Project (GTEx) to evaluate differential expression. GSEA was used to assess the mRNA expression profiles.^71^ The hallmark gene set (h.all.v7.2.symbols) and Kyoto Encyclopaedia of Genes and Genomes (KEGG) gene set (c2.cp.kegg.v7.2.symbols) were used for analysis. The GEPIA database was used to analyse gene expression.^72^

### 4.19 Statistical analysis

Statistical analyses were performed using SPSS and GraphPad Prism 7.0 software. Student's *t*‐test, log‐rank test or Mann–Whitney *U* test were used for comparisons of two groups, and one‐way ANOVA was used to analyse experimental data of three or more groups. Chi‐squared test was used to analyse the relationships between SLC25A21‐AS1 expression and clinicopathological characteristics. Numerical data are expressed as the means ± SD. The differences were considered statistically significant at *p < .05, **p < .01, and ***p < .001. NS corresponded to non‐significant differences.

## CONFLICT OF INTEREST

The authors declare that there is no conflict of interest that could be perceived as prejudicing the impartiality of the research reported.

## FUNDING INFORMATION

This work was supported by the Special Research Fund for Central Universities, Peking Union Medical College (Grant no. 3332020093), the National Key R&D Program of China (Grant no. 2021YFF1201303, 2020AAA0109505 and YS2021YFF120009), the CAMS innovation Fund for Medical Sciences (CIFMS) (Grant no. 2021‐1‐I2M‐012) and the R&D Program of Beijing Municipal Education Commission (Grant no. KJZD20191002302).

## Supporting information

Figure S1 Effects of other fatty acids on cell proliferation of ESCC cells and the effect of palmitic acid (PA) on tumour growth
Figure S2 The filtration of *SLC25A21‐AS1* and expression level
Figure S3 Validation of the efficiency of *SLC25A21‐AS1* overexpression by RT‐qPCR in KYSE30 and KYSE450 cells
Figure S4 The relationship between *SLC25A21‐AS1* and Myc target gene expression
Figure S5 The efficiency of knockdown SLC25A21 expression and its effect on ESCC cells
Figure S6 The expression of *SLC25A21‐AS1* between tumour and adjacent normal tissues
Figure S7 The CD36 expression and effect of HFD on ESCC cell lines. Figure S8 The PA/HFD affect mTOR/STAT3 pathway and regulated *SLC25A21‐AS1* transcript.

Table S1 The gene primers sequence
Table S2 The shRNA and siRNA sequence of SLC25A21‐AS1, SLC25A21 and NPM1
Table S3 The full‐length sequence of lncRNA SLC25A21‐AS1 full‐length in ESCC cell lines KYSE30. (1703bp)
Table S4 The predicted binding peaks of transcriptional factors STAT3 at the SLC25A21‐AS1 promoter region by JASPAR
Table S5 The clinicopathological characteristics were stratified by hyperlipidaemia in 38 ESCC patients
