# Supplementary material for: Lipid metabolism–related lncRNA SLC25A21‐AS1 promotes the progression of oesophageal squamous cell carcinoma by regulating the NPM1/c‐Myc axis and SLC25A21 expression
Source: Clin Transl Med. 2022 Jun 23;12(6):e944. doi: 10.1002/ctm2.944 (PMC9218933; doi:10.1002/ctm2.944)
Supplement: Supplementary file 1 — Figure S1 Effects of other fatty acids on cell proliferation of ESCC cells and the effect of palmitic acid (PA) on tumour growth Figure S2 The filtration of SLC25A21‐AS1 and expression level Figure S3 Validation of the efficiency of SLC25A21‐AS1 overexpression by RT‐qPCR in KYSE30 and KYSE450 cells Figure S4 The relationship between SLC25A21‐AS1 and Myc target gene expression Figure S5 The efficiency of knockdown SLC25A21 expression and its effect on ESCC cells Figure S6 The expression of SLC25A21‐AS1 between tumour and adjacent normal tissues Figure S7 The CD36 expression and effect of HFD on ESCC cell lines. Figure S8 The PA/HFD affect mTOR/STAT3 pathway and regulated SLC25A21‐AS1 transcript. [file CTM2-12-e944-s001.pdf]

Supplementary Figure and legend

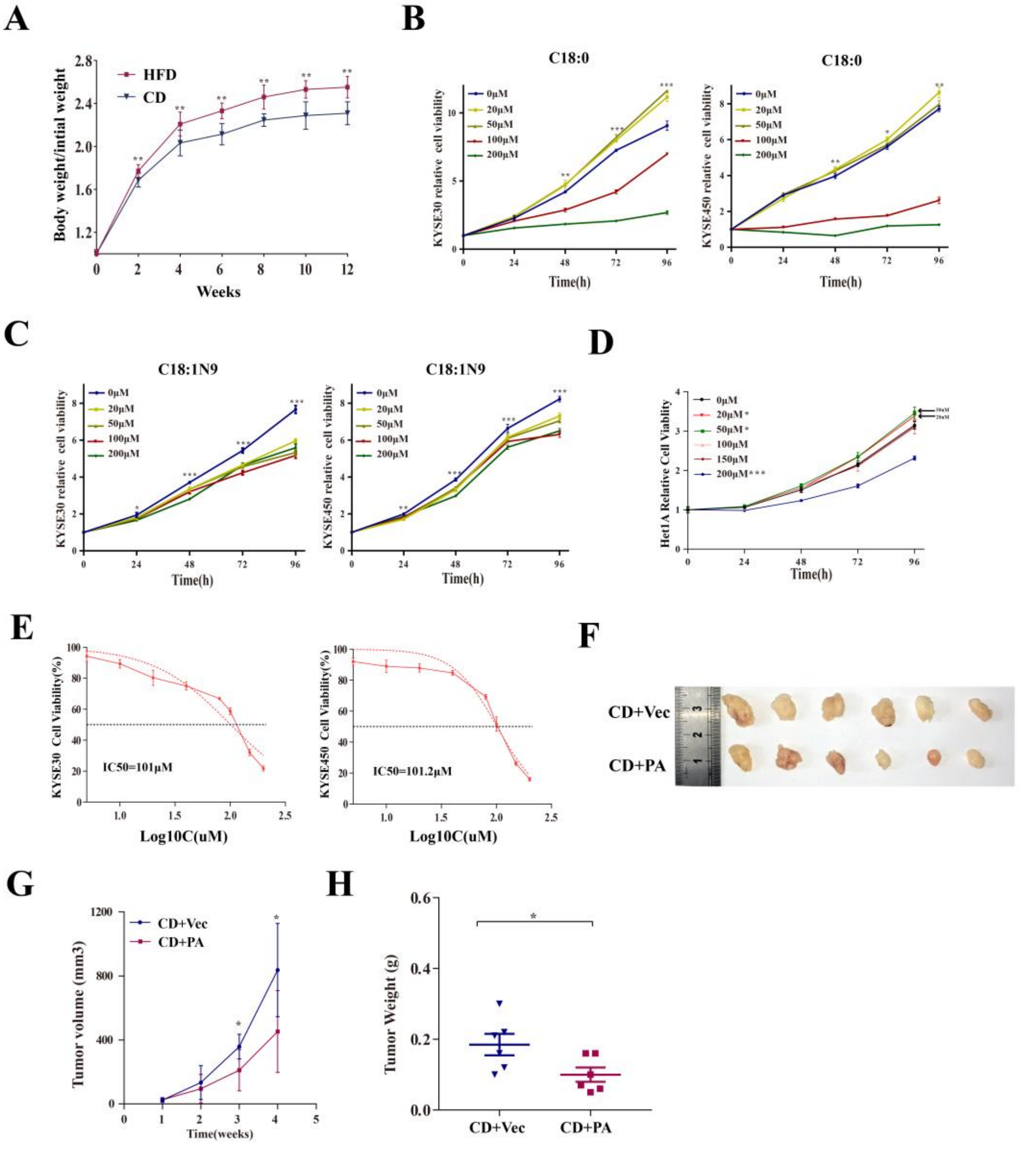

Supplementary Fig. 1 Effects of other fatty acids on cell proliferation of ESCC cells and the effect of palmitic acid (PA) on tumor growth.

A The average body weight was higher in high-fat diet (HFD) group than in control diet (CD) group. The average body weight was plotted normalizing data to the initial body weight. **B-C** Effect of stearic acid C18:0 and oleic acid C18:1N9 on cell proliferation of KYSE30 and KYSE450 cells at different concentrations (0-200 μM). **D** Effect of PA

on cell proliferation of Het1A cells at different concentrations (0-200 μM). **E** The half-maximal inhibitory concentration (IC50) analysis of PA in KYSE30 and KYSE450 cells. **F** The images and **G-H** volumes and weights of the xenograft tumors established in CD-fed mice with PA (10.26mg/kg/day). The BALB/cA-nu mice were treated with PA (10.26 mg/kg/day) by gavage and started one week after injected KYSE30 cell line. The CD+Vec group received 2% fatty acid-free BSA daily.

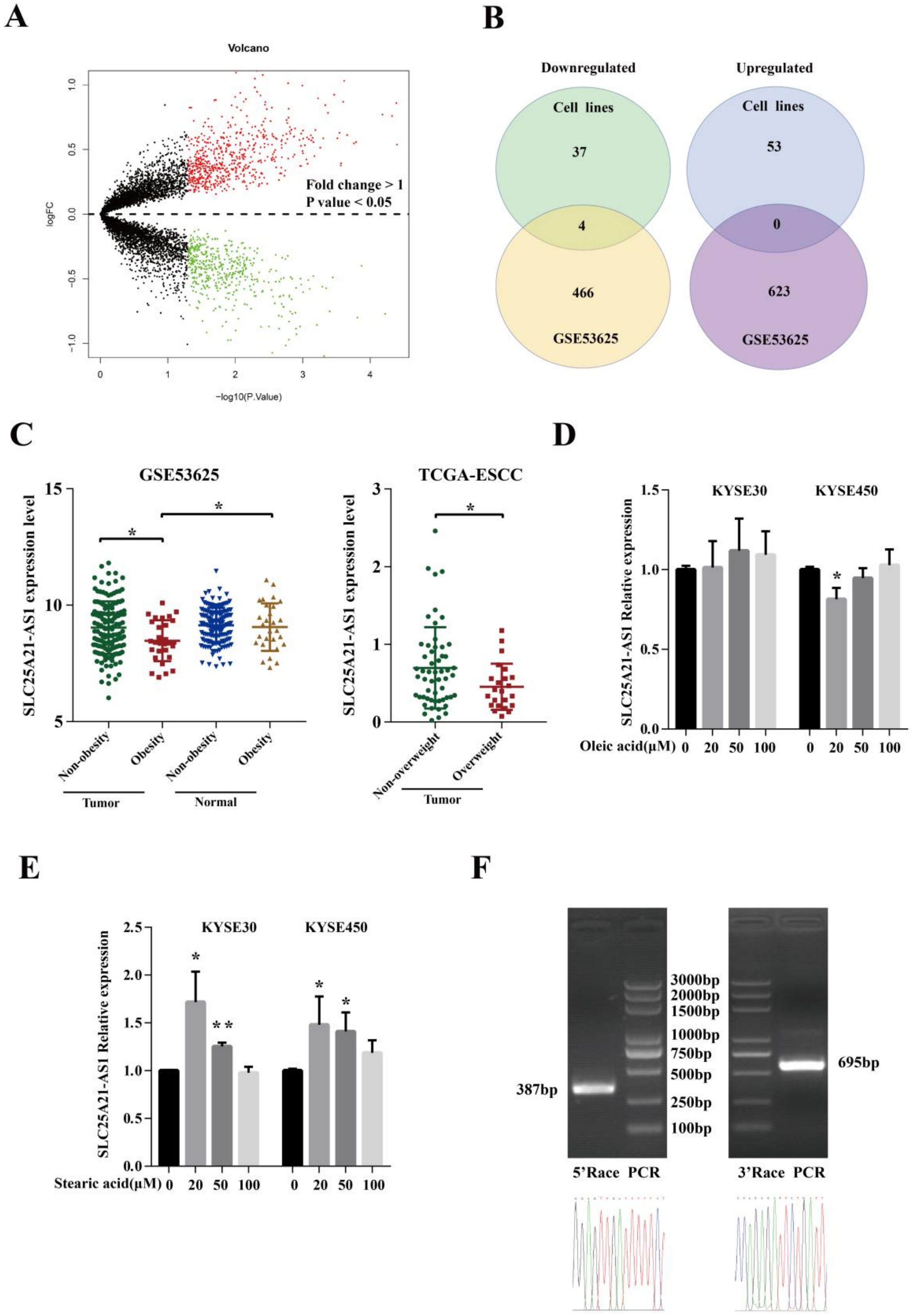

Supplementary Fig. 2 The filtration of *SLC25A21-AS1* and expression level.

A The volcano plot of the expression of differential lncRNAs in patients with or without hyperlipemia. **B** RNA-seq of *SLC25A21-AS1* stable-knockdown cells. The intersection

of upregulated (right) and downregulated (left) lncRNAs in cell lines and GSE53625 dataset, respectively. **C** The SLC25A21-AS1 expression level of patients with obesity (BMI > 28) or overweight (BMI:24-27.9) in GSE53625 and TCGA datasets. **D-E** *SLC25A21-AS1* expression in KYSE30 and KYSE450 cells treated with oleic acid and stearic acid at different concentrations (0-100 μM). **F** 5'RACE and 3'RACE PCR electrophoresis of *SLC25A21-AS1* sequence and Sanger sequencing analysis.

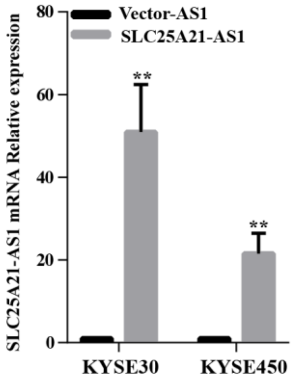

Supplementary Fig. 3 Validation of the efficiency of *SLC25A21-AS1* overexpression by RT-qPCR in KYSE30 and KYSE450 cells.

A

Accession	Protein name	Sequence coverge(%)	Score
P35527	KRT9	17.82	86.44
P35908	KRT2	11.27	72.56
P81605	DCD	10	58.49
E5RGW4	NPM1	15.25	36.49
Q4LE79	DSP	0.39	32
A0A087WTA8	COL1A2	0.81	31

B

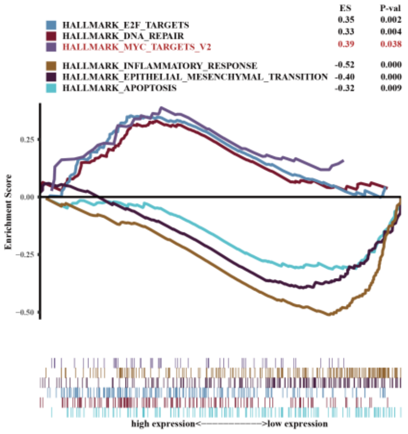

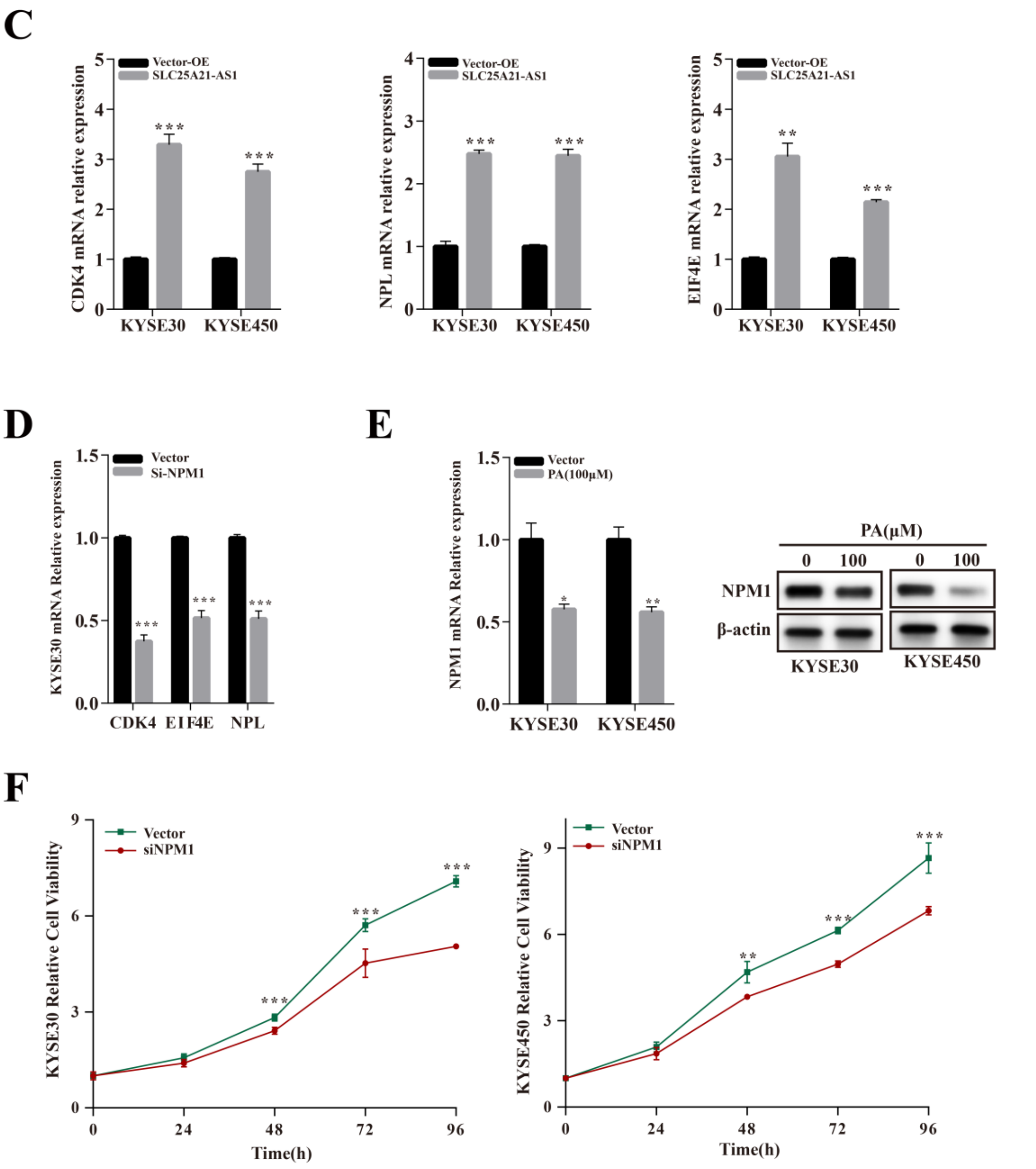

Supplementary Figure 4. The relationship between *SLC25A21-AS1* and Myc

target gene expression.

A *SLC25A21-AS1*-binding protein with the top matching scores identified by mass spectrometry after cutting off the gel. **B** GSEA function enrichment analysis of *SLC25A21-AS1* expression in GSE53625 dataset. **C** The expression of c-Myc target genes, CDK4, NPL, and EIF4E, in *SLC25A21-AS1*-overexpressing and vector ESCC cells as detected by RT-qPCR. **D** The expression of c-Myc target genes, CDK4, EIF4E, and NPL in NPM1-knockdown and vector control in KYSE30 cells. **E** Left panel shows the expression of NPM1 with or without 100μM PA by RT-qPCR. The right panel shows the protein expression of NPM1 with or without palmitic acid (PA, 100 μM) treatment in the ESCC cells. **F** Cell proliferation of NPM1-knockdown and vector in KYSE30 and KYSE450 cells.

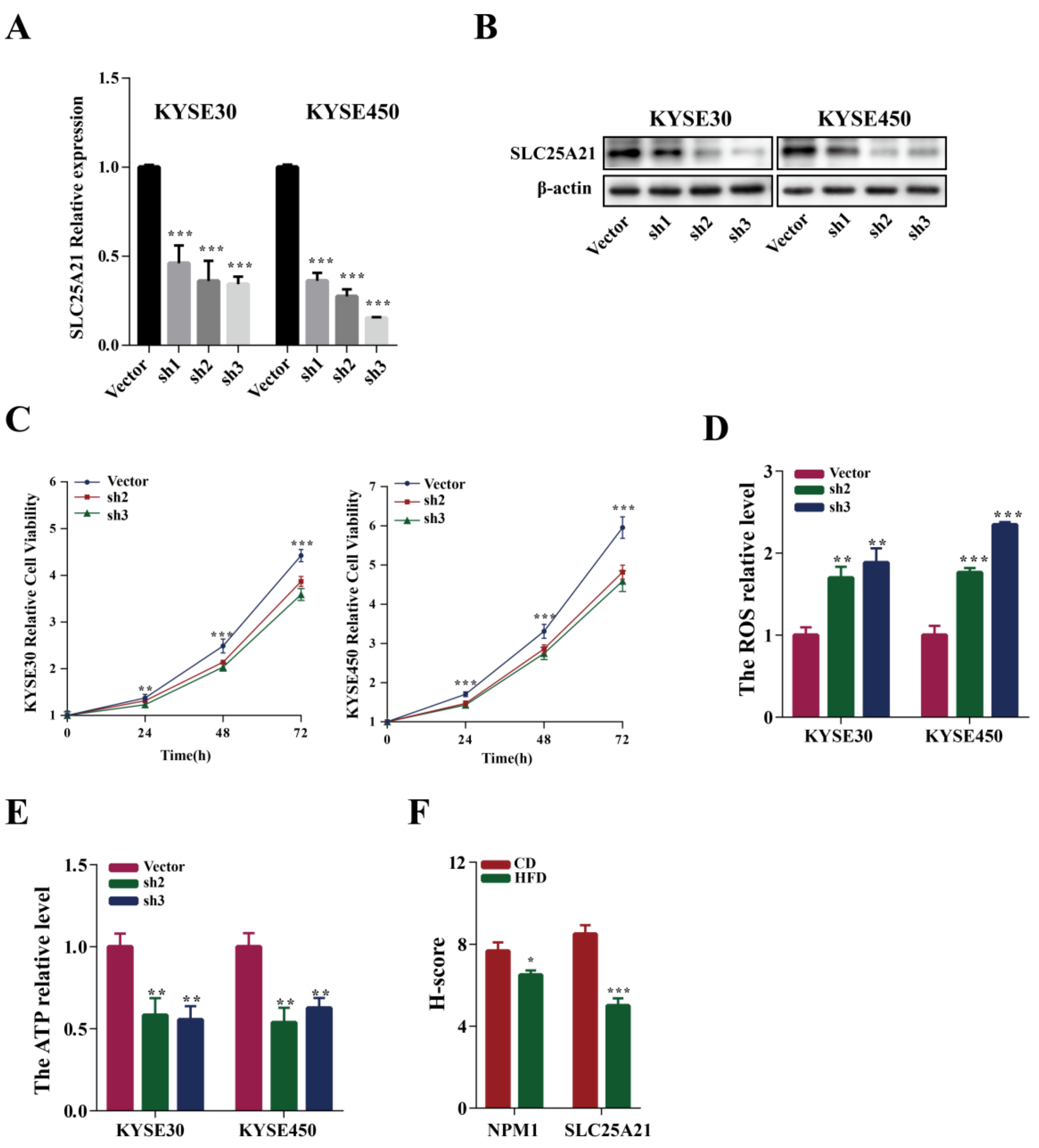

Supplementary Fig. 5 The efficiency of knockdown SLC25A21 expression and its effect on ESCC cells.

A Validation of the efficiency of knockdown SLC25A21 expression in KYSE30 and KYSE450 cells by RT-qPCR. **B** Validation of the efficiency of knockdown SLC25A21 expression in KYSE30 and KYSE450 cells by western blotting. **C** Cell proliferation of SLC25A21-knockdown and vector in KYSE30 and KYSE450 cells. **D** The ROS level of knockdown SLC25A21 expression and vector in KYSE30 and KYSE450 cell lines.

E The ATP level of knockdown SLC25A21 expression and vector in KYSE30 and KYSE450 cell lines. **F** The NPM1 and SLC25A21 protein expression results by immunochemistry assays. $p < 0.05$; ** $p < 0.01$; ***$p < 0.001$.

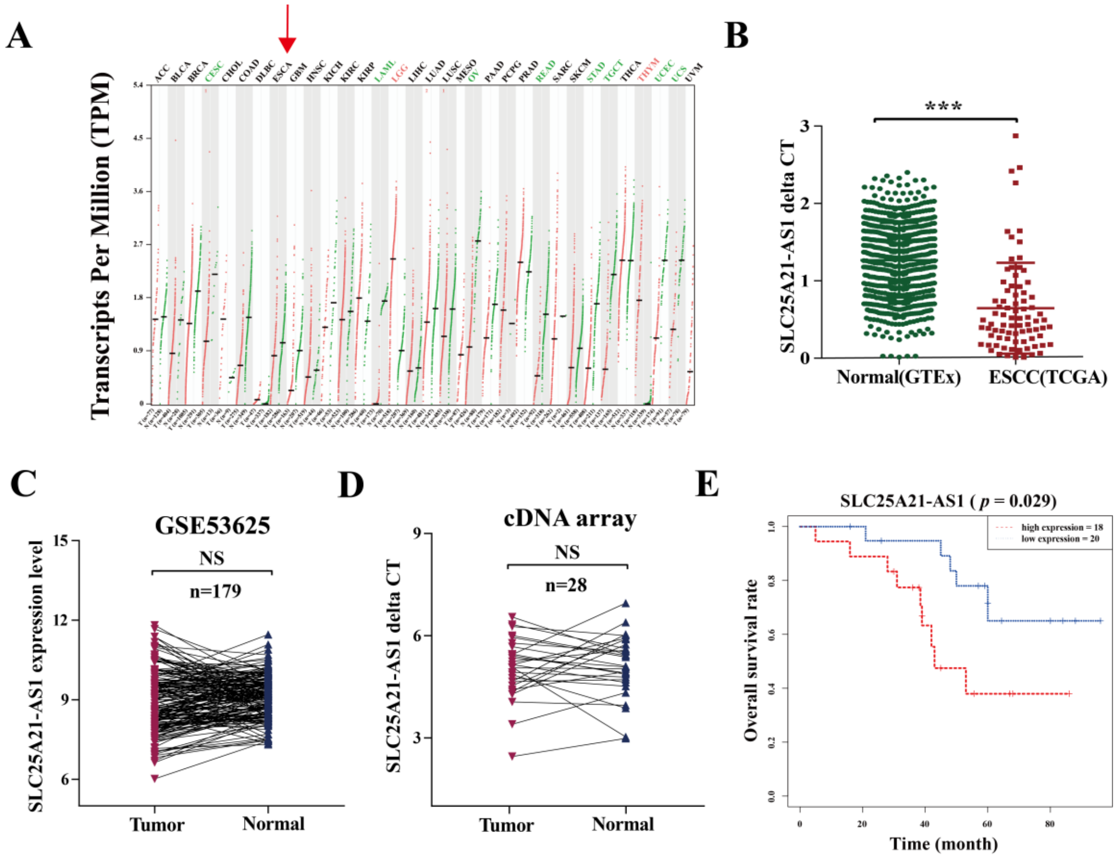

Supplementary Fig. 6 The expression of *SLC25A21-AS1* between tumor and adjacent normal tissues.

A The expression of *SLC25A21-AS1* in different tumor types from the GEPIA database. The red arrow is esophageal adenocarcinoma (ESCA) and ESCC. **B** The expression of *SLC25A21-AS1* is obtained from TCGA-ESCC, the normal esophagus tissues expression is obtained from GTEx database. **C** The expression of *SLC25A21-AS1* in 179 paired tumor and adjacent normal tissues from GSE53625 dataset. **D** The expression of *SLC25A21-AS1* in 28 paired tumorous and adjacent normal tissues belong

to cDNA microarrays by RT-qPCR. The delta CT value is negatively correlated with the expression level of *SLC25A21-AS1*. **E** The Kaplan-Meier survival curve showed that *SLC25A21-AS1* expression was associated with overall survival in 38 ESCC patients as another independent cohort.

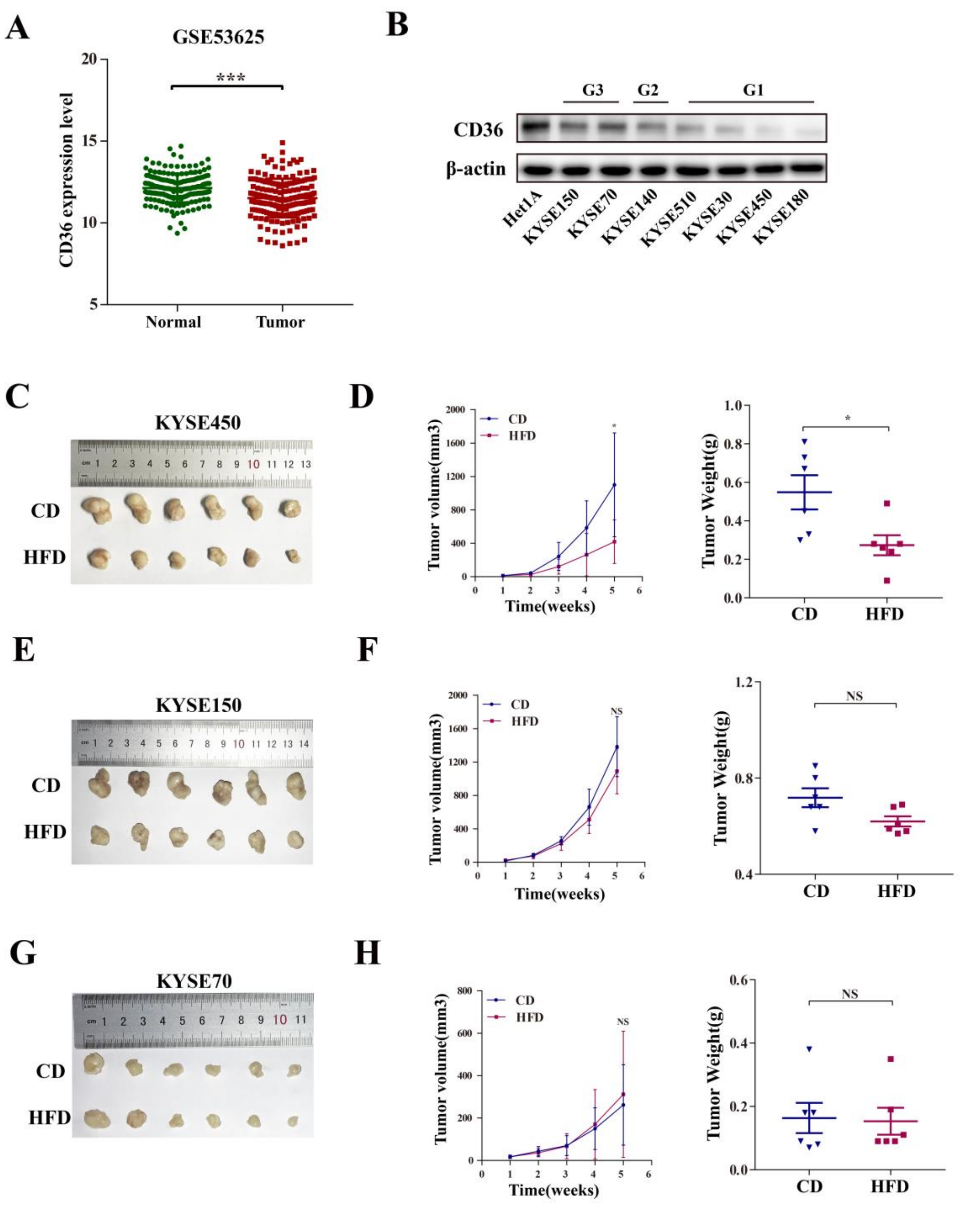

Supplement Fig. 7 The CD36 expression and effect of HFD on ESCC cell lines.

A The expression of CD36 was analysed in GSE53625 dataset. **B** The CD36 protein expression was analysed in Het1A, KYSE150, KYSE70, KYSE140, KYSE510, KYSE30, KYSE450 and KYSE180. **C** The images and **D** volumes and weights of the xenograft tumors established of KYSE450 in HFD and CD-fed mice. **E** The images and

F volumes and weights of the xenograft tumors established of KYSE150 in HFD and CD-fed mice. **G** The images and **H** volumes and weights of the xenograft tumors established of KYSE70 in HFD and CD-fed mice. $p < 0.05$; ** $p < 0.01$; ***$p < 0.001$.

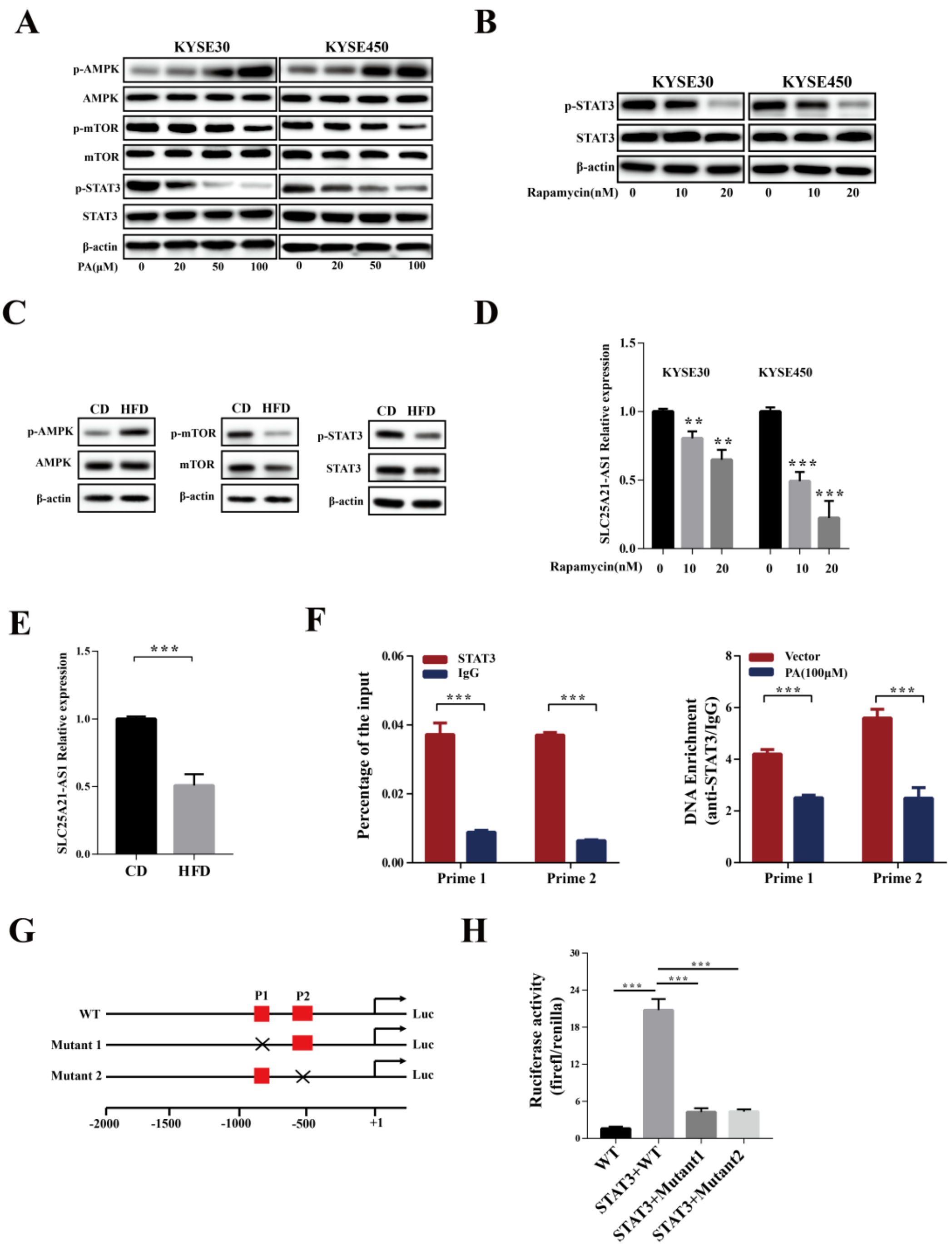

Supplement Fig. 8 The PA/HFD affect mTOR/STAT3 pathway and regulated *SLC25A21-AS1* transcript.

A Increased protein expression of p-AMPK and decreased expression of p-mTOR, p-STAT3 in KYSE30 and KYSE450 cells treated with palmitic acid (PA). **B** Decreased expression of phosphorylated STAT3 in KYSE30 and KYSE450 cells treated with rapamycin at different concentrations (0, 10 and 20 nM). **C** *SLC25A21-AS1* expression was downregulated in KYSE30 and KYSE450 cells treated with rapamycin by RT-qPCR. **D** Decreased protein expression of p-STAT3, p-mTOR and increased expression of p-AMPK in high-fat diet (HFD) group, compared with the common diet (CD) group. **E** *SLC25A21-AS1* expression was decreased in HFD group compared with the CD groups as detected by RT-qPCR. **F** ChIP-qPCR assay using an anti-STAT3 showed that STAT3 bound to the promoter region of *SLC25A21-AS1* in KYSE30 cells (left panels), and the binding ability was reduced when treated with 100 μM PA 24h prior to the ChIP assay (right panels). Primers specific for JASPAR predicting the binding peaks were used for ChIP-qPCR. **G** Schematic representation of the mutant construction of the SLC25A21-AS1 promoter in the dual-luciferase assay. “P1 and P2”, the prime region, “WT”, wild type SLC25A21-AS1 promoter. “X”, deletion mutation region. **H** Relative luciferase activities of reporters containing the SLC25A21-AS1 promoter or mutants 48h after co-transfection with the STAT3 overexpression plasmid or vector(Vec). Firefly luciferase activity was normalized to the control Renilla luciferase activity. $p < 0.05$; ** $p < 0.01$; ***$p < 0.001$.
