# Supplementary material for: Lipid metabolism–related lncRNA SLC25A21‐AS1 promotes the progression of oesophageal squamous cell carcinoma by regulating the NPM1/c‐Myc axis and SLC25A21 expression
Source: Clin Transl Med. 2022 Jun 23;12(6):e944. doi: 10.1002/ctm2.944 (PMC9218933; doi:10.1002/ctm2.944)
Supplement: Supplementary file 2 — Table S1 The gene primers sequence Table S2 The shRNA and siRNA sequence of SLC25A21‐AS1, SLC25A21 and NPM1 Table S3 The full‐length sequence of lncRNA SLC25A21‐AS1 full‐length in ESCC cell lines KYSE30. (1703bp) Table S4 The predicted binding peaks of transcriptional factors STAT3 at the SLC25A21‐AS1 promoter region by JASPAR Table S5 The clinicopathological characteristics were stratified by hyperlipidaemia in 38 ESCC patients [file CTM2-12-e944-s002.pdf]

Supplementary Table

Supplementary Table 1. The gene primers sequence.

Gene	Primers sequence
β-Actin-F	CATGTACGTTGCTATCCAGGC
β-Actin-R	CTCCTTAATGTCACGCACGAT
18s-F	ACACGGACAGGATTGACAGA
18s-R	GGACATCTAAGGGCATCACA
SLC25A21-AS1-F	GCCAACCCAAACCCATCC
SLC25A21-AS1-R	CGCAGCCTGCACAGCCTACT
SLC25A21-OL-F	ACATTTTCGGAGCCGAGGG
SLC25A21-OL-R	TACTGATCCAGAGAGCCCCG
SLC25A21-N-OL-F	TATCTTGGCTGAAACCCCAAAAA
SLC25A21-N-OL-R	CAGTGACACATATCCCAGCAAT
EIF4E-F	GAAACCACCCCTACTCCTAATCC
EIF4E-R	AGAGTGCCCATCTGTTCTGTA
NPL-F	GGTGGTCGTTTCCCCAACAAA
NPL-R	GCCAGGTGTGGTAACTGCT
CDK4-F	ATGGCTACCTCTCGATATGAGC
CDK4-R	CATTGGGGACTCTCACACTCT
NPM1-F	GGAGGTGGTAGCAAGGTTCC
NPM1-R	TTCACTGGCGCTTTTTCTTCA
DHTKD1-F	CCTTCCACACGGCAGGATTAT
DHTKD1-R	GGTAAACGTCTCCTTTTGCAGT
GCDH-F	CGTCCCGAGTTTGACTGGC
GCDH-R	GATGCGAGGCATGAGTCTCT
GAPDH-F	GGACCTGACCTGCCGTCTAG
GAPDH-R	GTAGCCCAGGATGCCCTTGA
U99-F	CCTCCTTTTCTTGGCGGGGA
U99-R	CGTTTGAGGATAGAACCAGC

Supplementary Table 2. The shRNA and siRNA sequence of SLC25A21-AS1, SLC25A21 and NPM1.

ID	Sequence(5'-3')
NC-shRNA(SLC25A21-AS1)	AAACGTGACACGTTCGGAGAACGA ATTCTCCGAACGTGTCACGTTT
shSLC25A21-AS1-2	GCAACTAAGTCAAAGCAAATCCGA AGATTTGCTTTGACTTAGTTGC
shSLC25A21-AS1-3	GGTTGTGAACCACTGACTTAACGA ATTAAGTCAGTGGTTCACAACC
NC-shSLC25A21	TTCTCCGAACGTGTCACGT
shSLC25A21	GCTTTGTACAAAGGCCTGCTT
shSLC25A21-1	CCTCAGTCATTAACATCCCTT
siNPM1-1	GGAGGAAGAUGCAGAGUCATT
si-NC	UUCUCCGAACGUGUCACGUTT

Supplementary Table 3. The full-length sequence of lncRNA SLC25A21-AS1 full-length in ESCC cell lines KYSE30. (1703bp).

AATCAACTCCCGGCCTCCTCTGCTCTCGCGCCTCTAGGGGCTCAAGCACTGCTCCGGGAGCGCTGAATTTTGAGGAAGAGGGCAGAACTTCA
GTAAGGAGACCTGTCTGGGCAACGGTTTCAGGACACGCGGTGGGGAAAGCGACTAGCCTCCGGCGGGGCAGGGCGGGCTGTCCTTACCTGCA
GAACCACCGGCCACGATCTGCCGAGAAGCCTCGCGCACTAAGCTGACTTCAGGCTTGGCGGACATCTTCGCCAGGCGGGAGGACAAGGGAGT
GGGCTGAGATGCGTCAACGAGCTCGCAGCCTGCACAGCCTACTGATCCAGAGAGCCCCGGCTGGGCTGGTCCTCAAGCGCGTTGGCTCCCTG
TGGAGCAGCAATCCGGCGACTGCTGGAAAGCGAGGGTTCGAGGCGCAGATTCGTCGCGCGATCTCCGGCGCGTCGGAACCTGTTCGCAGCGC
TCTCGCAGAGGCGCCCTCGGCTCCGAAAATGTCTCTGGAAAGAAGACCCGCGGATGGGTTTGGGTTGGCTCACCGCAGCGGCTCAGGCTTTG
TCTCCTAGATTGGCGCCCACGACCTTCTCTATCAGGAGCCTCCGGTAGTTTGTCAAACTAGACGGTTGCCTCTGATAACTCAAGTGAAGTGA
CCCAGGCCTGGAGCGCCAAGTACAAAGCGGGGACGACACTTGCACCTCCTGGCAAGCCAGAGCACAGTGAGGTTCAAAAGAAGGGACAAGTG
AATAAATTCCTCAGTTAGTTGTCCTATAATTGTGGTCTGTAGCAACTAAGTCAAAGCAAATCTAATTTACACTTACCTTACAATAGAGCTAA
GGTAGTACTTTACCAAAAATGGTTTTGATTTAAGGTAAAATCATACCTCATCAACTTGAGTTTTATTTACCTAAGGACCAAAAGAAGAAAAA
GCCTGGTTACAAATAGTTTCTTCTATACCCAGGTCAGTGGTTCTTAAAAGTGGTTTTAGTACCCACAGCATCAGCATAATTTGTGAATGTGT
TAGGAAGTCAAATTCGTGGGTCCCACCCCGACCTAAACGGAATCAGAATCTCTGGGGATAGGGCCCAGAAACGAGTTCTCTGTTGATTGTCA
TGAACTTTAAAGGTTGTGAACCACTGACTTAAACAATTGAGGAACATTAAGGAACTTAACAGTATATTCTCTCAATAAAATGTTTGATTATT
CTTACTAGCCCCTTAGGATAGTTACCAAAACAAAACTACTAATTTATACTCTTTTAAAACTACAAGTTGCCTGTGTTATGTAGGAGAGATAT
AAGATTGTGAAATTTTCCAAAAATACCTGCCACACCCCTAACAGACAGGAGATGATAAGAGTAAAAGAGAGAATTGGGAGGCTTTAGAAGCC
CTATTGTAATATTTCTTAAAACACATTATTTATTGCTAAATATATTTAGTGTGAGCCATGTAAAAATTGCCATTTTTGGAGGTCAAAACCGT
TGAATATCAGCAATTTAGTATGGTTCAATTTATGTGGTTTCTAATCAAATGTACAAAGTACAATAATTTGGTTTCATACTTGATTTTTTATT
TCTTAGGCTGAGTCATTAAAAGTTGATCAGTGATATATGGCAAAATTATTTAAAGTGCTTGAGGAATTGGGGAGACTTGCTGCTTATGTGAT
CAGATATACAAAAATAAAGTTTTTCATTTCGCAAAAAAAAAAAAAAA

The yellow is the overlapping region.

The red is the SLC25A21-AS1-sh2 sequence

The green is the SLC25A21-AS1-sh3 sequence

Supplementary Table 4. The predicted binding peaks of transcriptional factors STAT3 at the SLC25A21-AS1 promoter region by JASPAR.

Name	Score	Relative score	Start	End	Strand	Predicted sequence
STAT3	10.115	0.920094	1283	1293	-	CTTACAGGAAA
STAT3	9.01063	0.906714	1283	1293	+	TTTCCTGTAAG
STAT3	8.06417	0.895249	1550	1560	+	TTTATTGAAAG
STAT3	7.22481	0.88508	1457	1467	+	TTGTTTGAAAA
STAT3	6.75057	0.879335	2029	2039	-	TTTCTGAAAAC
STAT3	6.46002	0.875815	2016	2026	-	TTTCTTGGTAA
STAT3	6.09257	0.871363	2030	2040	+	TTTTCAGAAAT
STAT3	6.02454	0.870539	66	76	+	TTTATAAGAAT
STAT3	5.95913	0.869747	2030	2040	-	ATTTCTGAAAA
STAT3	5.77421	0.867507	1439	1449	-	CTTTTTGTAAG
STAT3	5.31113	0.861897	1759	1769	-	AATCTTAGAAA
STAT3	4.75162	0.855119	2016	2026	+	TTACCAAGAAA
STAT3	4.70986	0.854613	66	76	-	ATTCTTATAAA
STAT3	4.23652	0.848878	1180	1190	-	CAGACTGGAAG
STAT3	3.78361	0.843391	1507	1517	-	ATGACTAGAAA
STAT3	3.62583	0.84148	1332	1342	+	CTTTAAGAAAG
STAT3	3.11053	0.835237	1439	1449	+	CTTACAAAAAG
STAT3	3.0336	0.834305	1282	1292	-	TTACAGGAAAT
STAT3	2.66861	0.829884	1801	1811	-	CTGTGGGGAAA
STAT3	2.57907	0.828799	902	912	-	TTTTTGGGGAG
STAT3	2.37317	0.826305	1626	1636	-	TTTATTATAAA
STAT3	2.10836	0.823097	1782	1792	-	CTTTAGAAAAA
STAT3	1.8919	0.820474	323	333	-	CTACTAAAAAT
STAT3	1.7932	0.819278	26	36	+	TATCTTATAAA
STAT3	1.56948	0.816568	1626	1636	+	TTTATAATAAA
STAT3	1.55255	0.816363	2015	2025	-	TTCTTGGTAAA
STAT3	1.50982	0.815846	1642	1652	+	ATGACTGTAAG
STAT3	1.09115	0.810774	1550	1560	-	CTTTCAATAAA
STAT3	1.05603	0.810348	1759	1769	+	TTTCTAAGATT
STAT3	0.904911	0.808517	1583	1593	+	TTTCTGGTTAT
STAT3	0.712172	0.806182	1582	1592	-	TAACCAGAAAA
STAT3	0.5981	0.8048	596	606	-	ATGAAAGAAAA
STAT3	0.513577	0.803776	2002	2012	+	AAGTTTGAAAA
STAT3	0.475773	0.803319	753	763	-	GTTATTATAAT
STAT3	0.460331	0.803131	269	279	-	CTACTTGGGAG
STAT3	0.398892	0.802387	1600	1610	+	ATTATGGCAAT
STAT3	0.349969	0.801794	326	336	-	TTTCTACTAAA

>SLC25A21-AS1 promoter

TATATAAATCAACTATGCTATTTTTTATCTTATAAATATCCTAATTTGCATTTATATTAGTTACATTTATAAGAATATATGAT
GGAATCACACATATTTATGCCAAATACTTATCACTCAACAAAACAGCATAAGTAGGATCAGAGTTTGATCTACTTTTTTTTT
TTGAGACCGAGTCTCGCTCTGTCGCCAGGTTGGAGTGCAGTGGCACGATCTCGGCTCACTGCAACCTCCGCCTCCTGGGTT
CAAGCGATTCTCCTGCCTCAGCCTCCCAAGTAGCTGGGACTGTAGCCGGACACCACCAAGCCCAGCTGATTTTTGTATTTTT
AGTAGAAACGGGGTTTCACCATGTTGGGGAGGATGGTCTCGATCTCTTGACCTGGTGATCCGCCTGCCTCCGCCTCACAAA
GTCCTGGGATTACAGGCGTAAGACACCGTGCCTGGCCGAGTATGATCTACTTTTAACATTCTTTGAGTTTAGCTGTGCCTAA
TACATAGTATGATCATATAAATTAGCATACAGGTGCTTATGAGTCATCAGTCTTAGCTTAGGATGTTAATCAATTGGCCTCA
CTTTCTCTTTGGACTAGCTGAGTTTTCTTTCATCAGGGTCATAGACTACAAATCAACGAGTCAGGTACAGAAGACAGAACC
TTAGGCATTGCGGAATTTGACCAAAAATGGTAAATAAATTGGTGCAAATTCATCATCACAATTGGAAAACTGAAAGAGCA
AGCAGTGGGGCCACTGCAATTATAATAACGGGTGGTGGCTCACGCCTGTAGTCCCACCACTTTGGGAGGTCAAGGGGGTC
AGATTGCTTGAACCCAGGCGTTGGAGACCAGCCTGGACAACATGGTGAAACCCTGACTCTACAAAAAAAAAAATCCCCTT
CCCCCACCTCCCCAAAAAACTAGCCGGGCATGGTGGTGGGCTCCTGTAGTCCCAGCTACTCTGGAAGCTGCAGTGAGAGG
ATCACTTGAGCCCGGGAGGTTGAGACCAGCCTGGGCAACATGGCGAAAGCCCGTCTCTACAAAAAAAATTAAAAAAAAA
AATAGCCGGCATGGTGATGCGCATCGGTAATTCCAGCTACTCAGGAGGCTGAGATGGGAGGATTGCTTGAACCCGGGAGT
TGGAGCAGCGAGCCGAGATCGCGCCATGGCAATCCAGCTCCGGTGCCTTCCAGTCTGGGCAACAGAGAGAGACTCCATCG
AAAAGAAAAAGGGGGGAGGGGAGGGGAGGGGAGGGGAGGGGTGGGGAGGGGAGGGGAAAGATTTTACAATTTCCTGTA
AGCAATGAATGGCTCTAATGTGATATTGATGAAATACCATCTTTAAGAAAGTGTACAGATTGTAAGTGACACGTGGCTGCT
TACGTTACTCCTGAGATGCAAGTAGATCATGACATAATGATAACCAGTCATAATTTGCAGTAATCCCTTACAAAAAGTCCA
AATTTGTTTGAAAATACAGAAACTTTTACTAGTCGAAGTTAAACATTAAACATTTTCTAGTCATTGCCTTAACAGGATTTAT
TAGAGTTACTATAATTTATTGAAAGAATGGCTAGATTTATAAAATGTTTTCTGGTTATGAGCCAATTATGGCAATTATTTTA
ATTATATATTTATAATAAAAATGCATGACTGTAAGATAAAATTTTAAACCAAATCATAATTTATATTTTAAAATAGCTTTAA
AATACTTGCACATAACACATGTCGTGGTTAGTAACTTTCTCCAGCTACATTTAAAAGTATTTCTAAGATTAATTACAACATT
TTTTTCTAAAGGTTTATTTTTTCCCCACAGCATAACTCTATAAATTAACGTGAGCCCATTATATCAAAGGTGTTTATGTGTGT
TACATATGCATTTAATTTTAATATCCTTATTTAAGGCACGAATATCCCCAAGCAAGCACAGTATGCAATAAGAAGTCCTGT
GGAGTCCTGTAAGTACTTTGATGAGGAATGCAGGATTTATGTAAGACCTGAGCGATAAGTTTGAAAATGTCTTTAAGAAAG
TAGTTTTCAGAAATTTTGCTAACTTGATAAAGGCTTTTGAGCAATATCTTTCAGACTTTTTACTAATTAAATAG

Prime1 :F:5’-GGAGGGGAGGGGAAAGATTTTACA-3’
R: 5’-GCAGCCACGTGTCACTTACA-3’

Prime2: F: 5’-CTGCTTACGTTACTCCTG-3’
R: 5’-AAGGCAATGACTAGAAAAT-3’

Supplementary Table 5. The clinicopathological characteristics stratified by hyperlipemia in 38 ESCC patients.

Variables	Hyperlipemia		Total	*p*-value
	No	Yes		
Age				0.136
<60	12(48.00%)	3(23.07%)	15	
≥60	13(52.00%)	10(76.92%)	23	
Gender				0.029*
Female	8(32.00%)	9 (69.23%)	17	
Male	17(68.00%)	4(30.77%)	21	
Smoking				0.016*
Yes	14(56.00%)	2(15.38%)	16	
No	11(44.00%)	11(84.62%)	22	
Drinking				0.429
Yes	11(44.00%)	4(30.76%)	15	
No	14(56.00%)	9(69.23%)	23	
Tumor grade				0.976
Well	5(20.00%)	3(23.07%)	8	
Moderately	12(48.00%)	6(46.15%)	18	
Poorly	8(32.00%)	4(30.76%)	12	
T stage				0.436
T1	2(8.00%)	1(7.69%)	3	
T2	5(20.00%)	5(38.46%)	10	
T3	15(60.00%)	7 (53.84%)	22	
T4	3(12.00%)	0(0.00%)	3	
N stage				0.129
N0	8(32.00%)	4(30.76%)	12	
N1	5(20.00%)	5(38.46%)	10	
N2	10(40.00%)	1(7.69%)	11	
N3	2(8.00%)	3(23.07%)	5	
TNM stage				0.532
I	1(4.00%)	1(7.69%)	2	
II	7(28.00%)	5 (38.46%)	12	
III	17(68.00%)	7(53.85%)	24	
BMI				0.092
Yes	5(20.00%)	6(46.15%)	11	
No	20(80.00%)	7(53.84%)	27	
Hypertension				0.019*

Yes	3(12.00%)	6(46.15%)	9	
No	22(88.00%)	7(53.84%)	29	
Hyperglycemia				0.203
Yes	3(12.00%)	4(30.76%)	7	
No	22(88.00%)	9(30.76%)	31	

*p <0.05;** p <0.01; *** p <0.001
